# Superbinder based phosphoproteomic landscape revealed PRKCD_pY313 mediates the activation of Src and p38 MAPK to promote TNBC progression

**DOI:** 10.1186/s12964-024-01487-z

**Published:** 2024-02-12

**Authors:** Yujiao Deng, Zhanwu Hou, Yizhen Li, Ming Yi, Ying Wu, Yi Zheng, Fei Yang, Guansheng Zhong, Qian Hao, Zhen Zhai, Meng Wang, Xiaobin Ma, Huafeng Kang, Fanpu Ji, Chenfang Dong, Huadong Liu, Zhijun Dai

**Affiliations:** 1 Department of Gastroenterology, The Second Affiliated Hospital of Xi’an Jiaotong University, Xi’an, China; 2 Department of Oncology, The Second Affiliated Hospital of Xi’an Jiaotong University, Xi’an, China; 3 Center for Mitochondrial Biology and Medicine & Douglas C. Wallace Institute for Mitochondrial and Epigenetic Information Sciences, The Key Laboratory of Biomedical Information Engineering of Ministry of Education, School of Life Science and Technology, Xi’an Jiaotong University, Xi’an, Shaanxi China; 4 Department of Breast Surgery, The First Affiliated Hospital, College of Medicine, Zhejiang University, Hangzhou, China; 5 Department of Infectious Diseases, The Second Affiliated Hospital of Xian Jiaotong University, Xi’an, China; 6 Department of Pathology and Pathophysiology, Department of Colorectal Surgery and Oncology, Key Laboratory of Cancer Prevention and Intervention, The Second Affiliated Hospital, Ministry of Education, Zhejiang University School of Medicine, Hangzhou, China; 7 Zhejiang Key Laboratory for Disease Proteomics, Zhejiang University School of Medicine, Hangzhou, 310058 China

**Keywords:** Breast cancer, Phosphoproteomic, Kinase activity, PRKCD, Src

## Abstract

Phosphorylation proteomics is the basis for the study of abnormally activated kinase signaling pathways in breast cancer, which facilitates the discovery of new oncogenic agents and drives the discovery of potential targets for early diagnosis and therapy of breast cancer. In this study, we have explored the aberrantly active kinases in breast cancer development and to elucidate the role of PRKCD_pY313 in triple negative breast cancer (TNBC) progression. We collected 47 pairs of breast cancer and paired far-cancer normal tissues and analyzed phosphorylated tyrosine (pY) peptides by Superbinder resin and further enriched the phosphorylated serine/threonine (pS/pT) peptides using TiO_2_ columns. We mapped the kinases activity of different subtypes of breast cancer and identified PRKCD_pY313 was upregulated in TNBC cell lines. Gain-of-function assay revealed that PRKCD_pY313 facilitated the proliferation, enhanced invasion, accelerated metastasis, increased the mitochondrial membrane potential and reduced ROS level of TNBC cell lines, while Y313F mutation and low PRKCD_pY313 reversed these effects. Furthermore, PRKCD_pY313 significantly upregulated Src_pY419 and p38_pT180/pY182, while low PRKCD_pY313 and PRKCD_Y313F had opposite effects. Dasatinib significantly inhibited the growth of PRKCD_pY313 overexpression cells, and this effect could be enhanced by Adezmapimod. In nude mice xenograft model, PRKCD_pY313 significantly promoted tumor progression, accompanied by increased levels of Ki-67, Bcl-xl and Vimentin, and decreased levels of Bad, cleaved caspase 3 and ZO1, which was opposite to the trend of Y313F group. Collectively, the heterogeneity of phosphorylation exists in different molecular subtypes of breast cancer. PRKCD_pY313 activates Src and accelerates TNBC progression, which could be inhibited by Dasatinib.

**Supplementary Information:**

The online version contains supplementary material available at 10.1186/s12964-024-01487-z.

## Introduction

Breast cancer is the most common malignant tumor in the world, with the highest morbidity and the second mortality among women [1]. Based on the histological status of estrogen receptor (ER), progesterone receptor (PR), human epidermal growth factor receptor 2 (HER2), and Ki-67, breast cancer can be divided into Luminal A, Luminal B, HER2-positive and triple-negative subtype [2]. At present, the application of targeted therapy in breast cancer is limited, especially in triple negative breast cancer (TNBC). Receptors, TKs, phosphatases, and proteases are all potential therapeutic targets [3, 4]. Kinase inhibitors account for most targeted cancer therapies, with 87% of them being tyrosine kinase inhibitors [5]. Abnormal kinase activity is an important feature of cancer cells, which regulates protein interaction networks and transforms signaling pathways, which, in turn, changes the biological behavior of cells [6]. Hence, studying the key molecules of the abnormally activated kinase signaling pathway can identify biomarkers of breast cancer [7]. Proteomics research is an important part of post-genome program, and phosphorylation is a common protein post-translational modification (PTM) [8, 9]. In cells, approximately one-third of proteins are phosphorylated, including serine, threonine, and tyrosine residues [10]. Mass spectrometry (MS) is currently the most effective method for PTM analysis [11, 12]. Due to the limitations of enrichment materials with high affinity, reliable specificity and strong anti-interference ability, significant challenges remain in the clinical translation of PTM [13]. Therefore, we chose novel Super-binder enrichment strategy to remove background interference from other phosphorylated peptides and improve the sensitivity of low-abundance phosphorylated tyrosine (pY) detection [14], then we systematically mapped the abnormal kinases of patients with breast cancer.

Protein kinase C (PKC) is a family of multifunctional serine/threonine kinases, consisting of a single peptide chain with a regulatory domain at the N-terminus, and a catalytic domain at the C-terminus. The PKC family includes 10 isoforms such as PRKCD, which can be activated by a variety of hormones, growth factors and neurotransmitters [15], and is involved in cell growth, differentiation and apoptosis. PRKCD is recognized as a bi-functional regulator of cell death and proliferation [16]. However, its role in breast cancer development is controversial [17–19]. PRKCD plays a dual role through autophagy, reducing the proliferation ability of breast cancer cells, which also regulating the self-renewal of cancer stem cells [20]. The Y313 site is in the hinge region, which may change the positional relationship between C2 and V5, and then change the protein activity and function [21, 22]. Further analysis showed PRKCD_pY313 expressed higher in TNBC cell lines than other subtype cells. Hence, we predicted that it may play a fundamental role in the progression of TNBC. Therefore, we evaluate the effect of PRKCD_pY313 on the progression of TNBC and the related kinase signaling, which could contribute to the precise treatment.

## Materials and methods

### Sample source and preparations

Cancer tissues and paired far-cancer normal specimens (more than 5 cm from the tumor) were collected from 47 patients with breast cancer [all females, married, Han nationality, aged 30–85 years, mean age (55 ± 12.25) years]. All included patients were histologically diagnosed with breast cancer and did not receive any treatment before breast surgery. After surgical resection and sampling by the pathology department, the specimens were rinsed, snap-frozen in liquid nitrogen, and stored at − 80 °C until use. The ischemic time at the time of all tissue collection was controlled to within 5 min to minimize degradation of post-translational modified proteins. Clinical characteristic data of all included patients are summarized in Table 1.

**Table 1:** Characteristics of included patients with breast cancer

Characteristics	Number
**Age, (Mean** ± **SD, years)**	55 ± 12.25
**Age distribution (years)**	
≥ 50	23
< 50	24
**Age at menarche (years)**	
≥ 14	25
< 14	22
**Menopause**	
Yes	31
No	16
**History of cancer**	
Yes	3
No	44
**Grade**	
G1	7
G2	28
G3	12
**T Stage**	
T1	10
T2	35
T3	2
**N stage**	
N0	28
N1	14
N2	3
N3	2
**Stage**	
IA	8
IIA	21
IIB	12
IIIA	4
IIIC	2
**Ki67 status**	
Low	11
Middle	9
High	27
**ER status**	
Positive	35
Negative	12
**PR status**	
Positive	32
Negative	15
**HER2 status**	
Positive	12
Negative	35

Abbreviations: *SD* standard deviation, *ER* Estrogen receptor, *PR* progesterone receptor, *HER2* human epidermal growth factor receptor 2, *T Stage* tumor stage, *N stage*, nodal stage

### Phosphopeptides enrichment and MS analysis

**Fig. 1:**
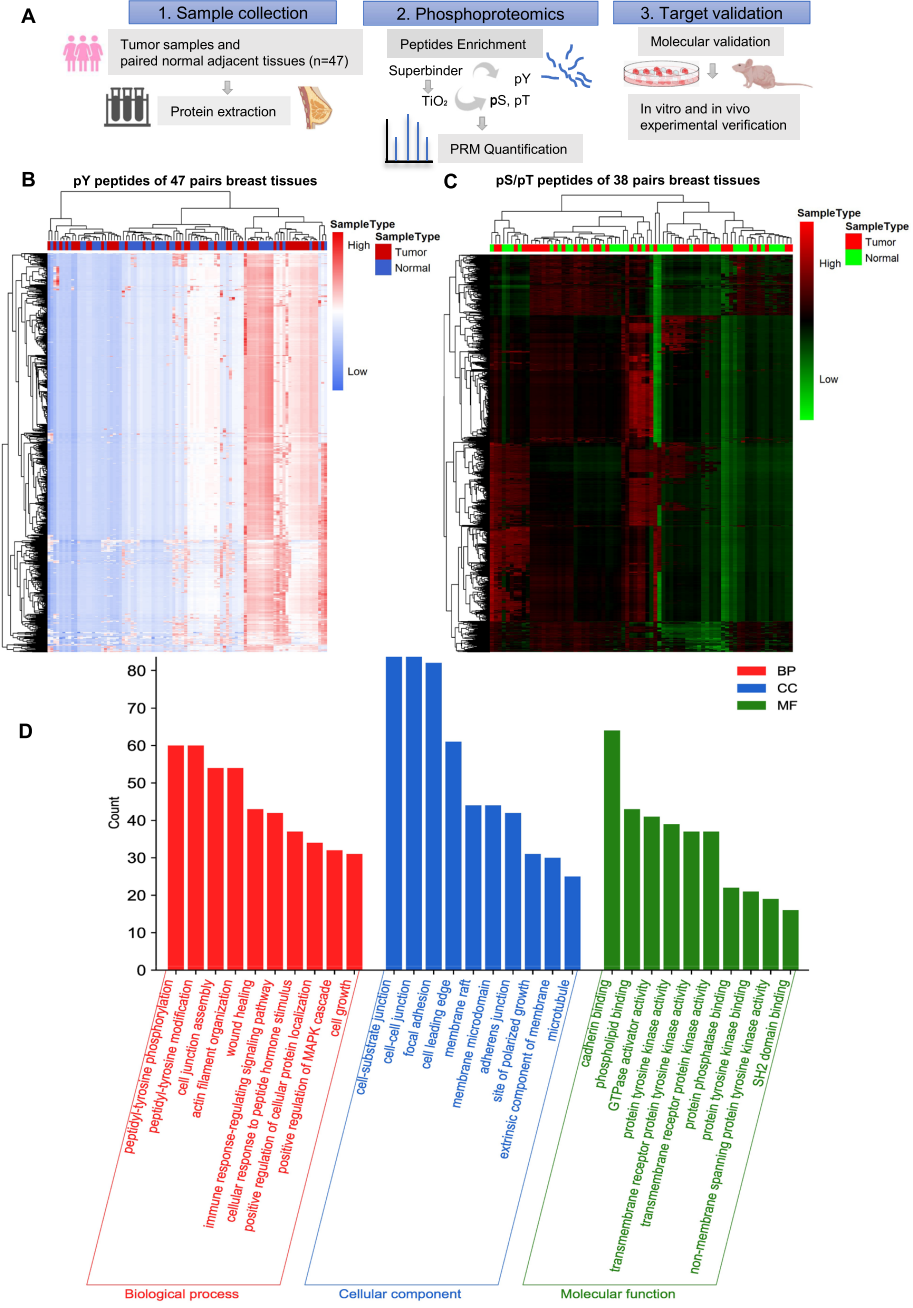
Phosphorylated peptides enrichment of breast tissues and further analysis. **A** The procedure of phosphorylated peptides enrichment and validation. **B** Heatmap of pY peptides in 47 pairs of breast cancer specimens and paired adjacent normal specimens. **C** Heatmap of pY peptides in 38 pairs of breast cancer specimens and paired adjacent normal specimens. **D** Barplot of Gene Ontology analysis of significant pY peptides related genes

The preparation procedures of MS sample has been described previously [23]. Total protein was extracted and digested by trypsin (Fig. 1A). After desalting with C18 column and resolving, the pY peptides were enriched with Superbinder resin, and phosphorylated serine/ threonine peptides (pS/pT) were enriched subsequently using TiO_2_ column. Due to tissue volume limitations, only 38 patients achieved subsequent enrichment of pS/pT peptides. Parallel reaction monitoring (PRM) quantification was applied through liquid chromatograph mass spectrometer (Q Exactive Plus). The database search was performed using the MaxQuant software. All data obtained in PRM mode were quantitatively analyzed using the Skyline software (version 3.7), and all target peptides were examined manually. The minimum peak area value of each sample was used to supplement the empty values.

### Cell lines and cell culture conditions

The human breast cancer cell lines (T47D, BT549, MCF7, MDA-MB-468, MDA-MB-231, and SKBR3) were purchased from Shaanxi Yike Biotechnology (Xi’an, China). MCF10A cells were cultured in DMEM/F12 (Procell) supplemented with 5% horse serum and 10 μg/mL insulin (Sigma). SKBR3 cells were grown in McCoy’s 5A medium (Procell) containing 10% fetal bovine serum (FBS). T47D and BT549 cells were cultured in a RPMI-1640 medium (Gibco) supplemented with 10% FBS. The MCF7, MDA-MB-231, and MDA-MB-468 cells were grown in DMEM (Gibco) supplemented with 10% FBS. All cells were incubated in a 5% CO_2_ incubator at 37 °C. Dasatinib (HY-10181) and Adezmapimod (HY-10256) were purchased from MedChemExpress (MCE, Shanghai, China). Phorbol 12-myristate 13-acetate (PMA) was purchased from Solabio (P6741).

### Lentivirus-mediated gene knockdown, overexpression and mutation

Gene knockdown or overexpression plasmid vectors were constructed, including short hairpin RNA (shRNA) targeting PRKCD in the pLKO.1 plasmid, PRKCD cDNA (PRKCD-OE) and point mutation at amino acid 313 (PRKCD_Y313F) in TK-pCDH-CMV-MCS-EF1-Puro (NC-TK), gRNA targeting PRKCD (PRKCD_KO) in lentivirus Crispr/Cas 9 (NC-sg). Nontargeting primers (NC-sh) was set as the control of the knockdown group. Lentivirus packaging and transfection were performed in accordance with the manufacturer’s instructions. All plasmid vectors were puromycin resistant, and stable clones were selected using puromycin (1 μg/mL, A1113803, Gibco) for 2 weeks. The primers used are shown in Tables S1–4.

### Protein extraction and western blot analysis

We extracted total proteins from tissues or cells for western blotting and MS analysis. The protein concentration was quantified using the Pierce BCA Protein Assay Kit (23,227; Thermo Fisher Scientific). All the antibodies used are summarized in Table S5.

### Cell proliferation and apoptosis assay

Cell proliferation (MDA-MB-231 and BT549) was tested using the MTT assay. Cells were seeded in the 12 well cell culture plates for 2 weeks, and colony formation rate was calculated. Cell apoptosis was detected using the Annexin V-APC/7-AAD or Annexin V-FITC/PI double staining cell apoptosis detection kit, and analyzed by flow cytometry (ACEA NovoCyte, USA).

### Wound healing assay

The cells with migration ability (MDA-MB-231 or BT549) were seeded in 12 well cell culture plates. When the degree of cells fusion close to 100%, a pipette tip (10 μL) was used to scratch the cells perpendicular to the culture plate, and the cells were washed twice with PBS in a gentle manner. The cells were then cultured in 1% serum medium and photographed at 0 h and 24 h, respectively. The percentage of cell wound closure was measured to quantify the wound healing.

### Cell invasion assay

We added 20% serum medium to the 8 μM pore non-coated polycarbonate chamber and seeded (1–2) × 10^5^ invasive cells (MDA-MB-231 or BT549) in 200 μL of serum-free medium in the chamber with the matrix above. After 24 h, the cells were fixed in methanol for 20 min and stained with 0.5% crystal violet for 30 min. We then removed the cells from the chamber gently, photographed and counted the invaded cells, and analyzed the results using the Image J software.

### Subcutaneous xenograft model

BT549 or MDA-MB-231 cells stable transfected with PRKCD_pY313 or PRKCD_Y313F overexpression (1 × 10^7^ cells) were suspended in 100 μL medium and implanted into female BALB/c-nude mice aged 4 weeks (GemPharmatech company, Naijing, China). We measured the tumor formation and mouse weight every 2–3 days until the endpoint. Tumor volume was calculated as 0.52× tumor length ×tumor width^2^. After reaching the endpoint, the mice were humanely sacrificed, and tumors were excised for further studies.

### Immunohistochemistry (IHC) analysis

We performed IHC analysis according to the staining procedure. The antibodies used are as follows: PRKCD (ab182126; Abcam, dilution 1:2000), Vimentin (Abmart, T55134, dilution 1: 200), ZO1 (Abmart, TA5145, dilution 1: 300), Bad (Abmart, T40052, dilution 1: 300), Bcl-xl (Abmart, T40057, dilution 1: 300), Ki67 (Servicebio, GB121141, dilution 1: 600).

### Dihydroethidium (DHE) probe labeling

The cells in good growth condition were digested and passaged in 12-well cell culture plates containing cell slides. Prepared DHE (S0063, Beyotime) staining solution was added and incubated at 37 °C for 30 min in the dark, then pictures were taken and recorded. DHE staining solution was removed and washed three times with precooled PBS at 4 °C. 200 μL reactive oxygenspecies (ROS) lysate was added for lysis on ice for 10 min. The supernatant was used for fluorescence detection and BCA quantification.

### Mito-tracker red/green probe labeling

Mito-tracker Red/Green CMXRos is a mitochondria-specific fluorescent probe whose staining of mitochondria is dependent on mitochondrial membrane potential (MMP). Therefore, the fluorescent probe can specifically label the biologically active mitochondria in living cells, observe the mitochondrial morphology, and reflect the changes of MMP. When the cell confluence reached 50 to 60%, the medium was discarded and washed three times with PBS. Mito-Tracker Red/Green staining solution was prepared in the dark, and the volume ratio of incomplete medium /Mito-Tracker was 10,000/1. 200 μL of prepared staining solution was added to each well and incubated at 37 °C for 30 min in the dark. Then we take pictures and record.

### Ethics statement

Experimental procedures were approved by the Ethics Committee of the Second Affiliated Hospital of Xi’an Jiaotong University and the written informed consent was obtained from all patients before enrolling in the research program. The in vivo assay was approved by the Institutional Animal Care and Use Committee of Xi’an Jiaotong University (Approved No. 2023–77).

### Software and statistical analysis

Heatmaps, Gene Ontology (GO) and Kyoto Encyclopedia of Genes and Genomes (KEGG) analysis were conducted by R Software (4.1.2). The protein networks were visualized using Cytoscape 3.7.1. All assays were repeated three times in vitro, and the data were analyzed using the GraphPad Prism 7. *P* value, *q* value (false discovery rate, FDR), log_2_ (fold change, FC), and Z-score were calculated using paired *t*-test to compare the paired tissue groups. Data are presented as mean ± standard deviation (SD). Unpaired Student’s *t*-test was used to compare the two groups, and one-way or two-way ANOVA with Dunnett’s multiple comparisons test was used to compare the multiple groups. Differences with *P* < 0.05 was considered as statistically significant.

## Results

### The enrichment of phosphorylated Tyr peptides by Superbinder and Ser/Thr enrichment by TiO_2_ columns

**Fig. 2:**
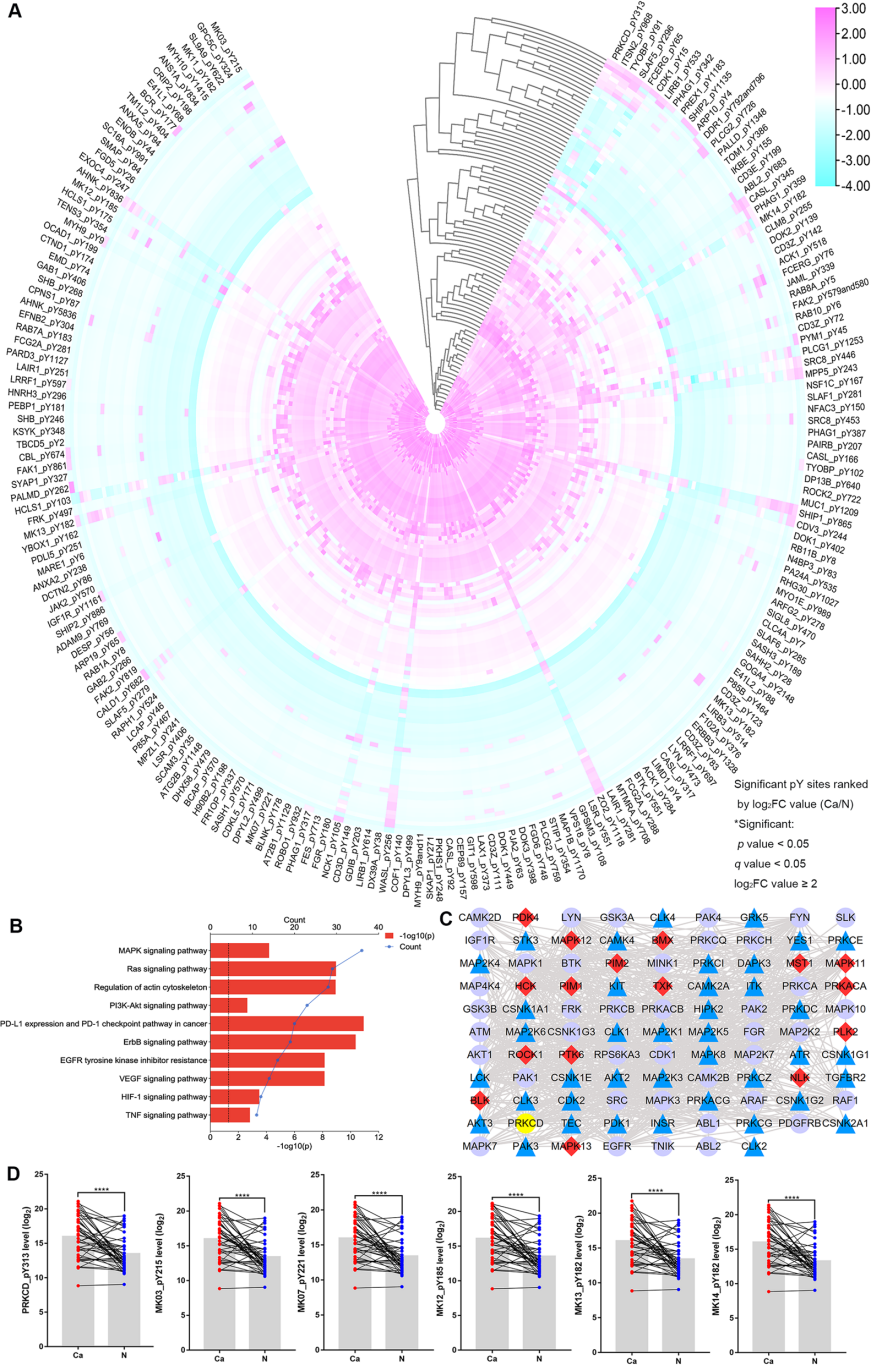
Significant phosphorylated tyrosine peptides in breast cancer tissues and functional analysis. **A** The significant pY peptides in 47 pairs of breast cancer specimens and paired adjacent normal specimens from mass spectrometry analysis, ranked by log_2_FC value. **B** Barplot of top 10 KEGG pathways of significant pY peptides related genes. **C** Intersection protein-protein interaction networks of PRKCD related genes among pY, pS or pT significant proteins in the mass spectrometry analysis. The triangle represents the substrates of PRKCD; the diamond represents the kinases of PRKCD; the circle represents the kinases and substrates of PRKCD. **D** The phosphorylated level analysis of PRKCD_pY313 and some of its related kinases (MAPK3_pY215, MAPK7_pY221, MAPK12_pY185, MAPK13_pY182, MAPK14_pY182) based on mass spectrometry data. pY, phosphorylated tyrosine; FC, fold change; KEGG, Kyoto Encyclopedia of Genes and Genomes. * *P* < 0.05, ** *P* < 0.01, *** *P* < 0.001, **** *P* < 0.0001

**Fig. 3:**
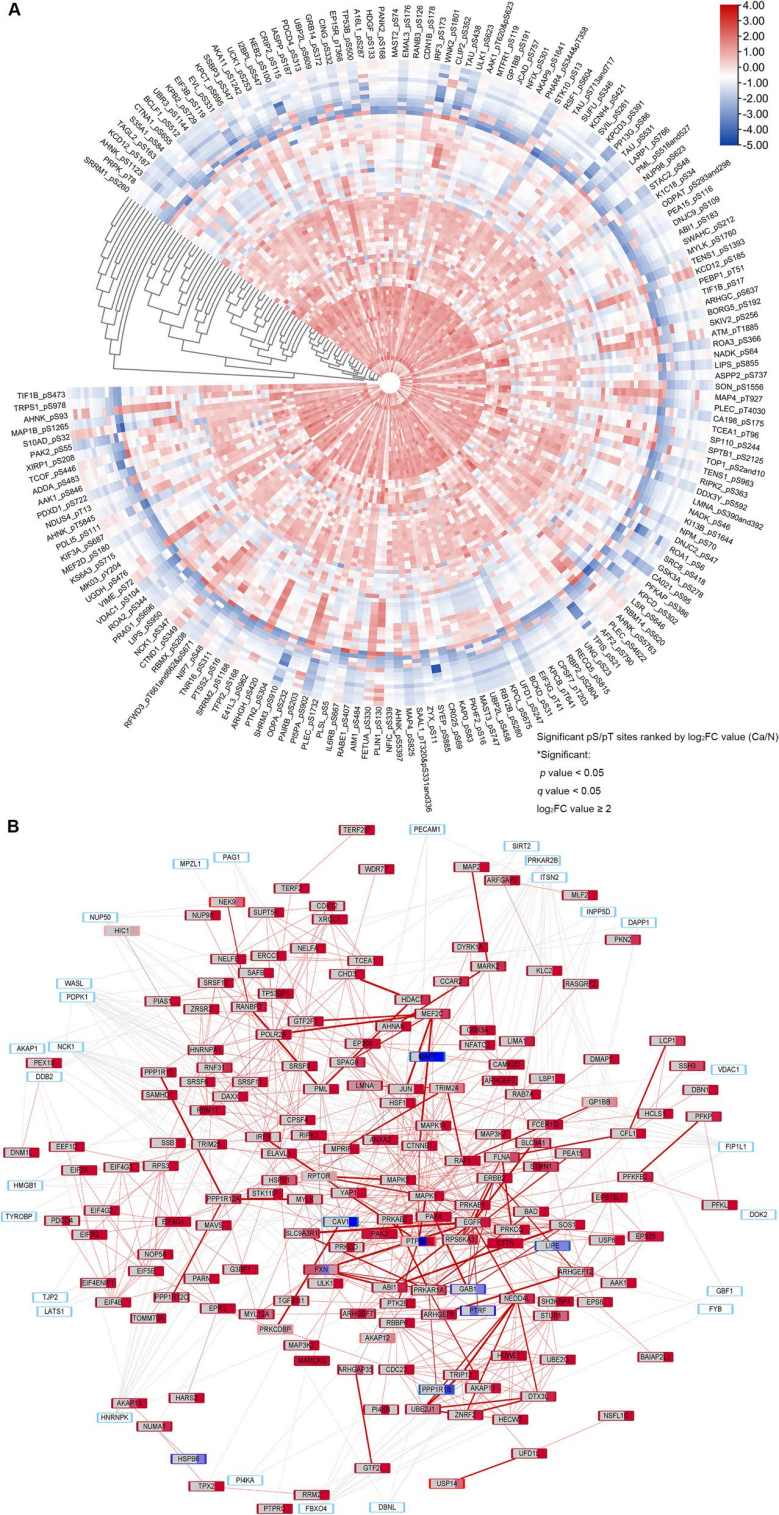
Significant phosphorylated serine/threonine peptides and personalized kinase map of patients with TNBC. **A** The significant pS/pT peptides in 38 pairs of breast cancer specimens and paired adjacent normal specimens, ranked by log_2_FC value. **B** The personalized kinase activity map of patient with triple-negative breast cancer based on the pY-pS/pT associated genes. Each protein frame is divided into four small squares, each of which represents one pY, pS or pT site. The color represents the log_10_(Ca/N) value, red represents the upregulation of this site, and the darker the color, the higher the upregulation ratio. Blue indicates downregulation, darker color indicates higher downregulation, and gray indicates no such site. The color of the border of each protein bar represents the sum of the log_10_(Ca/N) values of all sites of the protein. The thickness of the lines between the proteins indicates a test score ranging from 1 to 10, and the color of the lines ranging from gray to red indicates a test score ranging from 0 to 1. TNBC, triple-negative breast cancer; KEGG, Kyoto Encyclopedia of Genes and Genomes; FC, fold change; Ca/N, ratio of expression levels in breast cancer tumors to that in normal breast tissue

There were 1159 pY peptides of 551 proteins (Fig. 1B) and 2941 pS/pT peptides of 1320 proteins (Fig. 1C) were enriched in total. GO enrichment analysis of significant pY or pS/pT-related proteins was performed to understand their main functions (Figs. 1D and S1A). Overall, 188 significant pY peptides were captured in Fig. 2A. 281 pS/pT peptides of 159 proteins showed higher phosphorylated level in cancer than in normal tissues (Fig. 3A). To elucidate the individual kinase abnormal activity network, we mapped different subtypes of breast cancer patients (Figs. 3B and S2–4). In addition, most of pY and pS/pT peptides-related genes were clustered in the mitogen-activated protein kinases (MAPK) signaling (Figs. 2B and Fig. S1B). According to Netphorest predictions [24], 96 proteins interacted with PRKCD (Fig. 2C), including Src family and some kinases of MAPK signaling pathway. The top 20 genes of top 5 GO pathways of pY/pS/pT peptides in patients with breast cancer were listed in Figs. S5–7. And proteins enriched in top 5 KEGG pathways were shown in Figs. S8–13.

### PRKCD_pY313 expressed high in TNBC tissues and cells

**Fig. 4:**
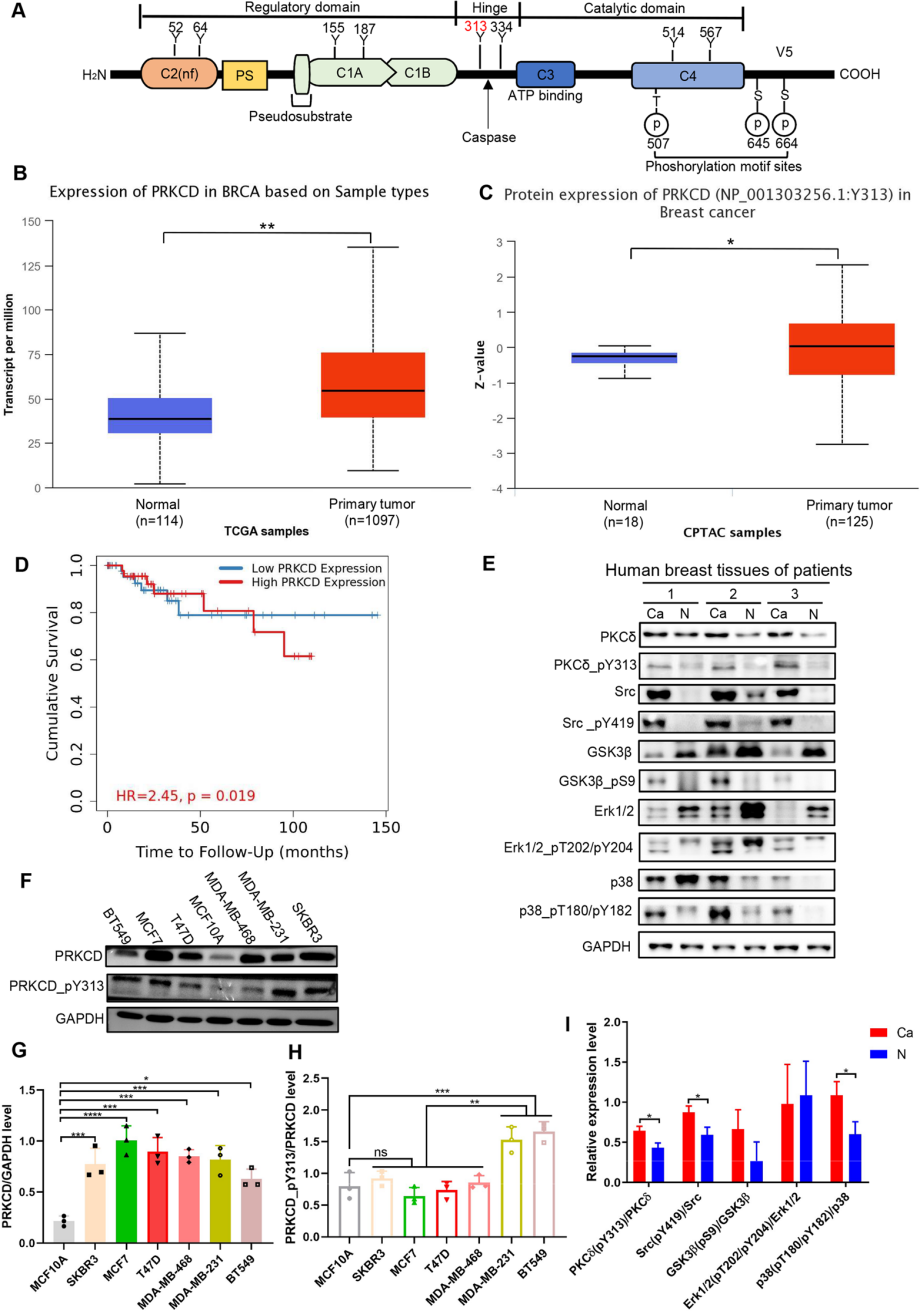
The expression level of PRKCD_pY313 in breast cancer tissues and cell lines. (**A**) The functional regions and tyrosine sites structure of PRKCD. (**B**) The mRNA level of PRKCD in breast cancer tissues based on TCGA samples. (**C**) Western blots revealed the protein expression level of PRKCD_pY313 in breast cancer tissues based on CPTAC samples. (**D**) Breast cancer patients with high PRKCD expression level has poor survival based on TCGA databases. (**E**) The level of PRKCD_pY313 and related kinases in breast cancer and paired far-cancer normal tissues. (**F**) The level of PRKCD_pY313 and PRKCD in breast cancer cell lines (BT549, MCF7, T47D, MDA-MB-231, SKBR3) and immortalized breast cells (MCF10A). (**G**) The relative expression of PRKCD after normalization to the signals of GAPDH in Fig. 4F. (**H**) The relative expression of PRKCD_pY313 after normalization to the signals of PRKCD in Fig. 4F. (**I**) The relative expression of the phosphorylation level of PRKCD_pY313 and its associated kinase active site after normalization to the signals of their protein in Fig. 4E. FC, fold change; nf, nonfunctional; TNBC, triple negative breast cancer. Mean ± SD, *n* = 3 per group, * *P* < 0.05, ** *P* < 0.01, *** *P* < 0.001, **** *P* < 0.0001

The difference of PRKCD level between cancer tissues and normal tissues was the largest, which accounted for 74.4% (35 pairs) of all included patients (Fig. 2A). As shown in the structure diagram, the Y313 site is in the hinge region of PRKCD (Fig. 4A). The levels of PRKCD and PRKCD_Y313 in breast cancer were validated in TCGA and CPTAC data (Fig. 4B-C). Kaplan-meier analysis showed that high PRKCD expression was associated with poor survival, disease specific survival and distant metastasis free survival of breast cancer patients, especially for TNBC (Figs. 4D and S14). According to MS and tissues data, PRKCD_pY313 and some phosphorylated level of kinases (Src and p38) were significantly higher in breast cancer tissues than in normal tissues (Figs. 2D, Fig. 4E, I). As for cell lines, PRKCD_pY313 was higher in TNBC cell lines (MDA-MB-231 and BT549) than other human breast cancer cells and normal breast cells (Fig. 4F-H). Therefore, we constructed stable TNBC cell lines expressed different PRKCD_pY313 level (Figs. S15A and 5F).

### PRKCD_pY313 accelerates the malignant biological behaviors of TNBC

**Fig. 5:**
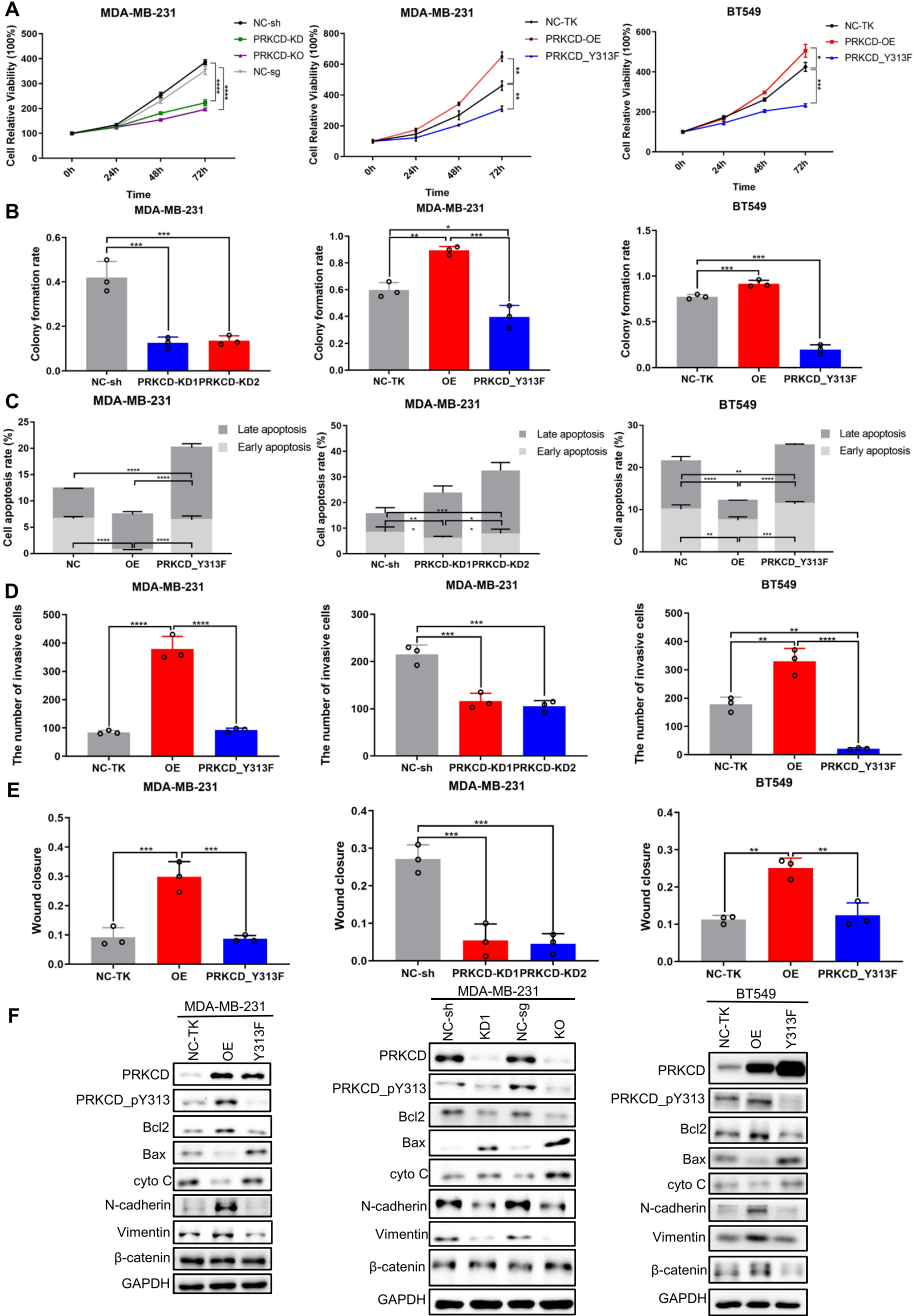
PRKCD_pY313 elevated the malignant biological behaviors of human TNBC cell lines. (**A**) The MTT assay in MDA-MB-231 and BT549 stable transfected TNBC cell lines with different PRKCD_pY313 level. (**B**) The colony formation rate of MDA-MB-231 and BT549 stable transfected cell lines with different PRKCD_pY313 level. (**C**) The apoptosis rate of MDA-MB-231 and BT549 stable transfected cell lines with different PRKCD_pY313 level, detected by flow cytometry. (**D**) The invasion assay of MDA-MB-231 and BT549 cell lines. (**E**) The quantification of wound healing assay of MDA-MB-231 and BT549 cell lines. (**F**) Western blot analysis of apoptotic related proteins (bcl-2, bax, cyto C), migration or invasion biomarkers (N-cadherin, Vimentin, β-catenin). All the results were presented from 3 independent experiments. TNBC, triple negative breast cancer; MTT, 3-(4,5)-dimethylthiahiazo (−z-y1)-3,5-di- phenytetrazoliumromide. Mean ± SD, *n* = 3 per group, * *P* < 0.05, ** *P* < 0.01, *** *P* < 0.001, **** *P* < 0.0001

In vitro experiments showed that high level of PRKCD_pY313 significantly enhanced MDA-MB-231 and BT549 cell proliferation (Fig. 5A) and colony formation ability (Figs. 5B and S15C), decreased the apoptotic rate of TNBC cells (Figs. 5C and S15B), accompanied by changes in apoptosis-related proteins, including Bcl-2, Bax and cyto C (Figs. 5F and S16A-C). We further assessed the effect of PRKCD_pY313 on TNBC cells migration and invasion by wound healing and transwell assays. As shown in Figs. 5D-E and S15D-E, compared with PRKCD_Y313F and PRKCD depletion group, PRKCD_pY313 overexpressing TNBC cells migrated and invaded faster, accompanied by upregulated with N-cadherin and Vimentin levels (Figs. 5F and S16D-F).

**Fig. 6:**
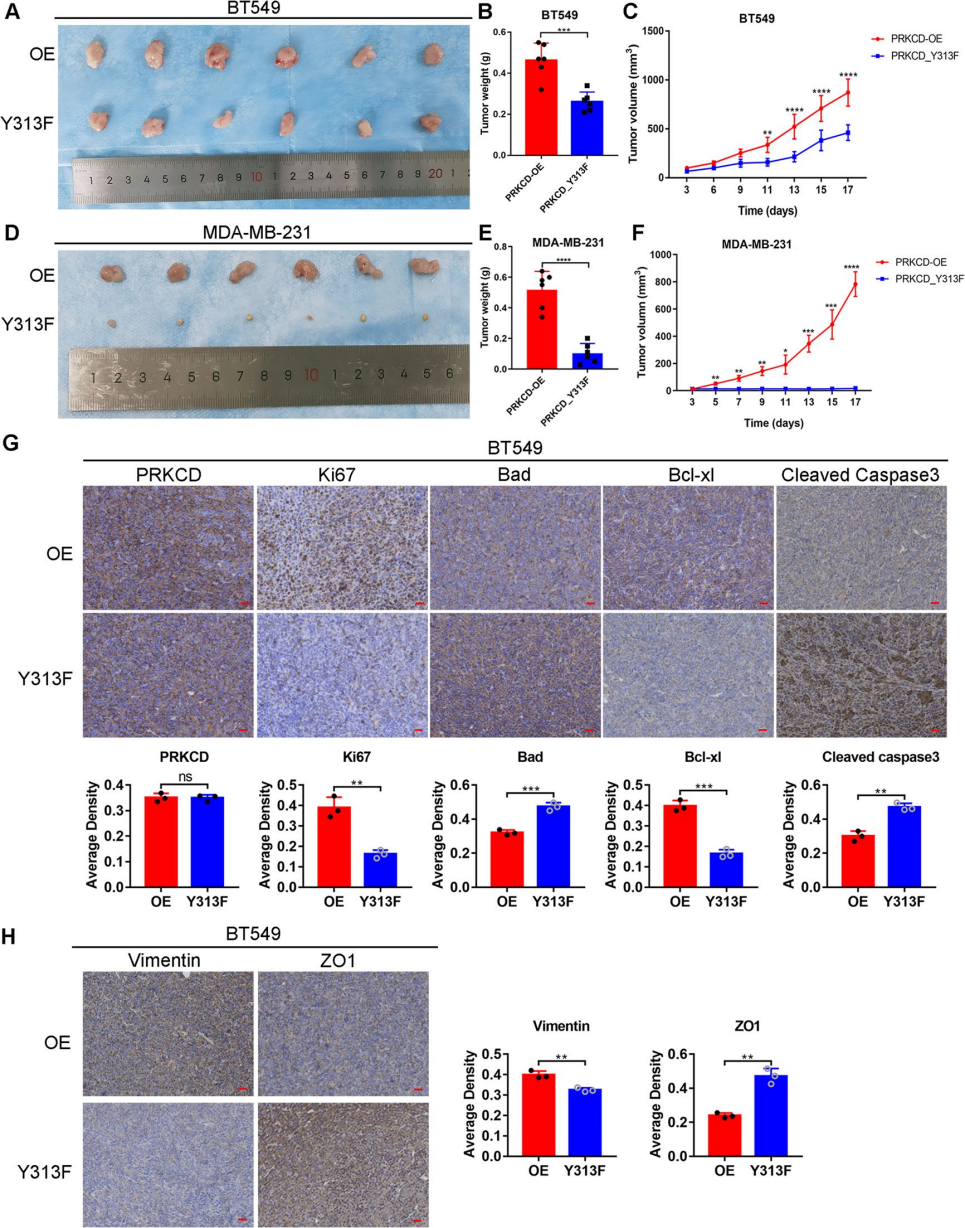
PRKCD_pY313 promoted the growth of tumor in the xenograft models. (**A**) Tumor volume of BT549 cells stably overexpressing PRKCD_pY313 or PRKCD_Y313F which was transplanted into BALB/c nude mice. (**B**) The tumor weights of BT549 cells stably overexpressing PRKCD_pY313 or PRKCD_Y313F which was transplanted into BALB/c nude mice. (**C**) The tumor growth curve of BT549 cells stably overexpressing PRKCD_pY313 or PRKCD_Y313F which was transplanted into BALB/c nude mice. (**D**) Tumor volume of MDA-MB-231 cells stably overexpressing PRKCD_pY313 or PRKCD_Y313F which was transplanted into BALB/c nude mice. (**E**) The tumor weights of MDA-MB-231 cells stably overexpressing PRKCD_pY313 or PRKCD_Y313F which was transplanted into BALB/c nude mice. (**F**) The tumor growth curve of MDA-MB-231 cells stably overexpressing PRKCD_pY313 or PRKCD_Y313F which was transplanted into BALB/c nude mice. Scale Bars = 25um. (**G**) IHC staining of PRKCD, Ki-67, Bad, Bcl-xl, and Cleaved caspase 3 in xenograft tumor samples transplanted with BT549 cells stably overexpressing PRKCD_pY313 or PRKCD_Y313F. (**H**) IHC staining of Vimentin and ZO1 in xenograft tumor transplanted with BT549 cells stably overexpressing PRKCD_pY313 or PRKCD_Y313F. IHC, immunohistochemistry. Scale Bars = 25um. Mean ± SD, *n* = 3 per group, * *P* < 0.05, ** *P* < 0.01, *** *P* < 0.001, **** *P* < 0.0001

In the xenograft models (Fig. 6A-F), transplated tumor of PRKCD_pY313 overexpressing TNBC cells showed larger volumes and higher weights than those of Y313F group. In IHC staining, the expression levels of Ki-67, Bcl-xl and Vimentin in PRKCD_pY313 overexpression group were higher than those in Y313F group, while the expression levels of Bad, Cleaved caspase 3 and ZO1 were downregulated (Fig. 6G-H). Taken together, these results reveal that PRKCD_pY313 promotes the proliferation, invasion and migration of TNBC cells.

### PRKCD_pY313 affects Src and p38 MAPK activity

**Fig. 7:**
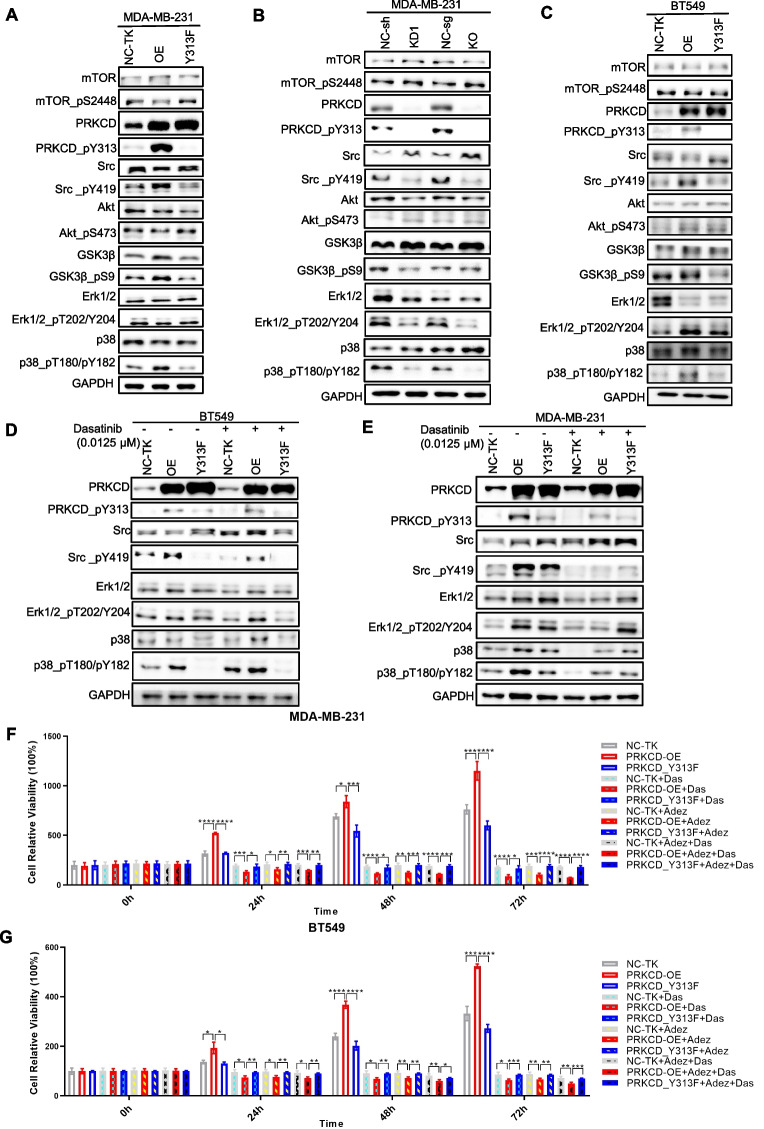
PRKCD related kinase activity in TNBC cell lines with different PRKCD_pY313 levels and changes after dasatinib treatment. (**A**) PRKCD-related kinases and their phosphorylation levels in MDA-MB-231 cells with different levels of PRKCD_pY313. (**B**) PRKCD-related kinases and their phosphorylation levels in MDA-MB-231 cells with PRKCD_pY313 knockdown and knockout. (**C**) PRKCD-related kinases and their phosphorylation levels in BT549 cells with different levels of PRKCD_pY313. (**D**) Kinases activity in BT549 cells with different PRKCD_pY313 level among dasatinib treatment. (**E**) Kinases activity in MDA-MB-231 cells with different PRKCD_pY313 level among dasatinib treatment. (**F**) The MTT assay of MDA-MB-231 cells with different PRKCD_pY313 level among 0.05 μM dasatinib and/or 20 μM Adezmapimod treatment. (**G**) The MTT assay of BT549 cells with different PRKCD_pY313 level among 0.05 μM dasatinib and/or 20 μM Adezmapimod treatment. All the results were presented from 3 independent experiments. Mean ± SD, *n* = 3 per group, * *P* < 0.05, ** *P* < 0.01, *** *P* < 0.001, **** *P* < 0.0001

According to a published study, IHC staining suggested that SFK_pY416 is related to phosphorylated CDCP1 and PRKCD_pY313 in TNBC cells, which provides a basis for its targeted therapy [18]. Further validation prompted that PRKCD_pY313 overexpression upregulated the Src_pY419 and p38_pT180/pY182, whereas PRKCD_Y313F and low PRKCD_pY313 group had the reversed effect (Fig. 7A-C and S17). We then treated the PRKCD_pY313 and PRKCD_Y313F overexpressed TNBC cells with Dasatinib, which could significantly and selectively downregulate Src_pY419 level to inhibit Src activity (Figs. 7D-E and S18).

Compared with PRKCD_Y313F, PRKCD_pY313 overexpressed cells responded more sensitively to Dasatinib, and its proliferation was effectively inhibited by 0.05 μM Dasatinib (Fig. S19). In addition, the inhibition effect of Dasatinib on cell proliferation activity was enhanced by 20 μM Adezmapimod (p38 inhibitor) treatment (Fig. 7F-G). Thus, PRKCD_pY313 affects Src activity and is sensitive to Src inhibitor Dasatinib.

### PRKCD_pY313 regulates ROS and MMP level

**Fig. 8:**
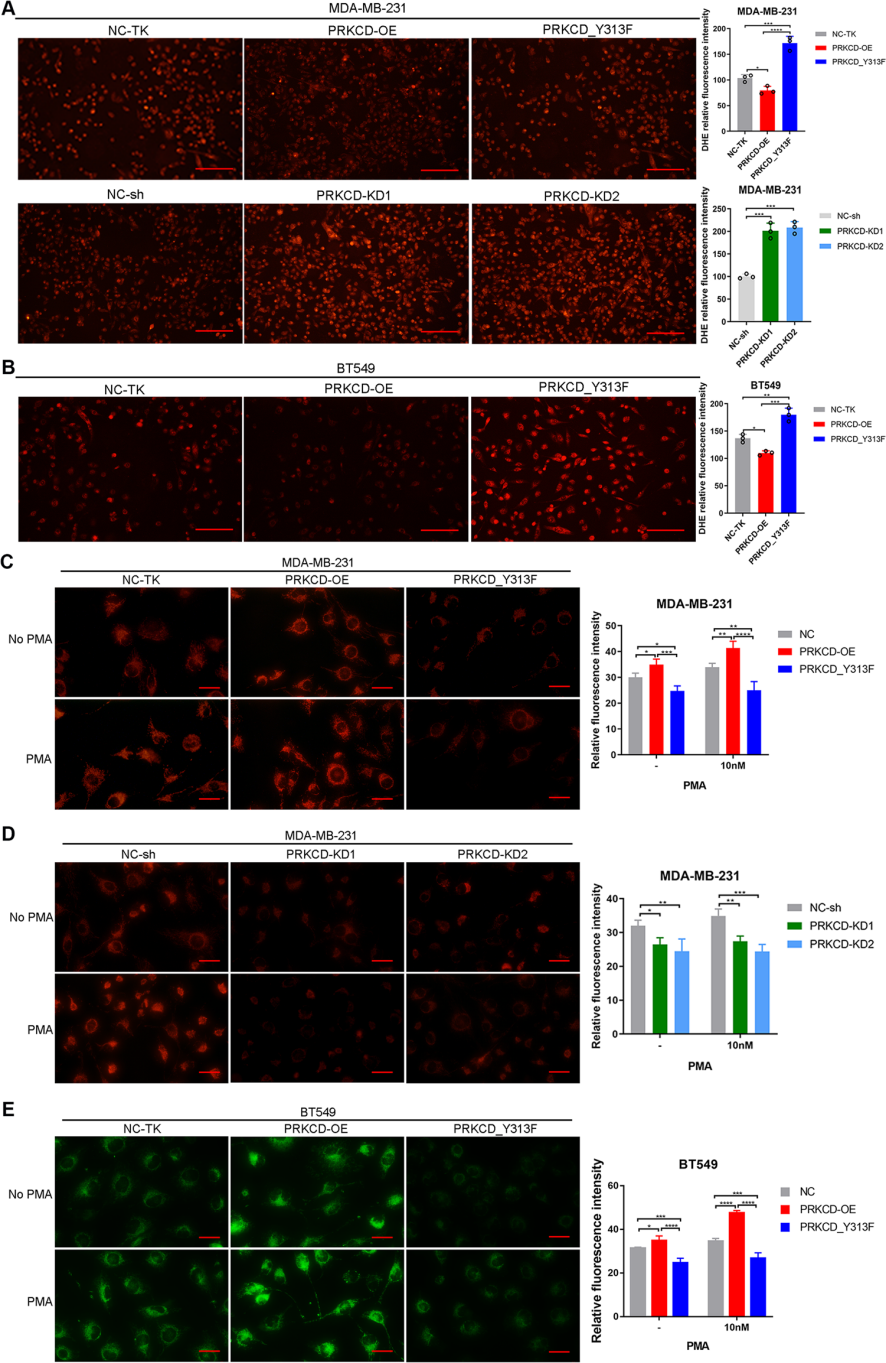
DHE probe labeling and mito-tracker staining in TNBC cell lines with different PRKCD_pY313 level. (**A**) DHE probe labeling in stable transfected MDA-MB-231 cell lines with different levels of PRKCD_pY313 and quantification. Scale Bars = 100um. (**B**) DHE probe labeling in stable transfected BT549 cell lines with different levels of PRKCD_pY313 and quantification, Bar = 100um. (**C**) Mitochondrial membrane potential intensity in stable transfected MDA-MB-231 cells with different levels of PRKCD_pY313 without PMA and after treatment with 10 nM PMA for 20 min. Scale Bars = 10um. (**D**) Mitochondrial membrane potential intensity in stable transfected MDA-MB-231 cells with low PRKCD_pY313 level without PMA and after treatment with 10 nM PMA for 20 min. Scale Bars = 10um. (**E**) Mitochondrial membrane potential intensity in stable transfected BT549 cells with different levels of PRKCD_pY313 without PMA and after treatment with 10 nM PMA for 20 min. Scale Bars = 10um. PMA, Phorbol 12-myristate 13-acetate; DHE, Dihydroethidium; TNBC, triple negative breast cancer. Mean ± SD, *n* = 3 per group, * *P* < 0.05, ** *P* < 0.01, *** *P* < 0.001, **** *P* < 0.0001

Previous research suggests that Src participates in mitochondrial dysfunction [25], and PRKCD may influence the intracellular level of ROS [26]. DHE probe labeling assay indicated that the ROS level of TNBC cells with low PRKCD_pY313 or PRKCD_Y313F was significantly higher than PRKCD_pY313 overexpressed group (Fig. 8A-B). Compared with the control group, the mitochondrial membrane potential of PRKCD_pY313 overexpression cells was significantly up-regulated, while that of PRKCD_Y313F cells was significantly down-regulated. The results were similar before and after PMA (PKC activator) treatment (Fig. 8C-E). Therefore, PRKCD_pY313 could increase the level of ROS and decrease the mitochondrial membrane potential in TNBC cells.

**Fig. 9:**
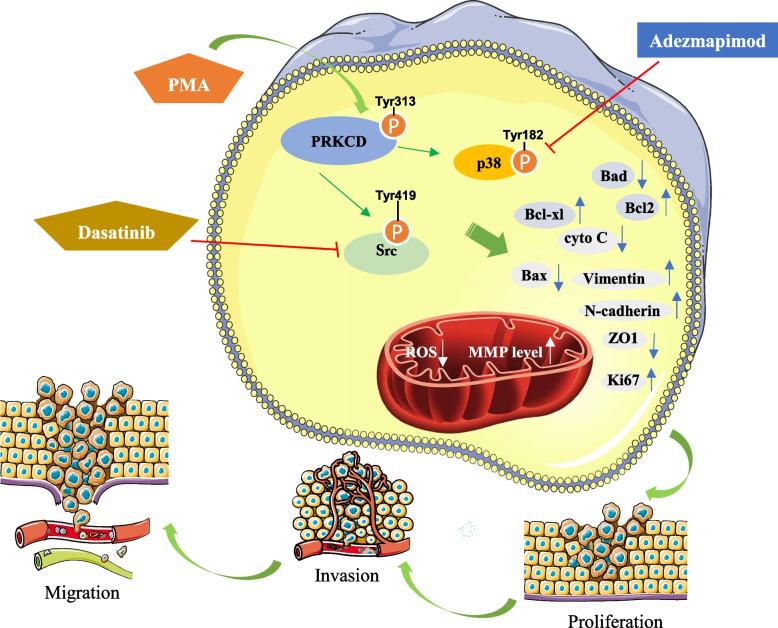
A proposed model to illustrate mechanisms and functions of PRKCD_pY313 mediates Src, p38 activity and regulates ROS, MMP, promoting TNBC progression

Here, we show that PRKCD_pY313 mediates Src and p38 activation, which occurs simultaneously with ROS reduction and MMP increase, and can promote the malignant biological behavior of TNBC cells. Dasatinib could inhibit this process, and the effect could be enhanced by Adezmapimod (Fig. 9).

## Discussion

Innovative, highly sensitive and high-throughput proteomics techniques contributed to accurately assess biomarkers in cancer development and evaluate the role of important proteins in cancer, thereby aiding in precise diagnosis and treatment [27]. The core challenge of phosphorylated proteomics is to enrich low abundance phosphorylated peptides in the proteome and detect highly sensitive means [28]. In this study, we used Superbinder beads to enrich pY sites of tyrosine kinase and to quantify intracellular tyrosine kinases. The newly established pY peptide enrichment strategy can remove background interference from other phosphorylated peptides and improve detection sensitivity. In addition, compared with the traditional pY enrichment method, Superbinder protein can be expressed in E.coli, and the preparation is simple, inexpensive, and has higher pY enrichment ability [14].

We further calculated and screened the MS data for key kinases, and validated it in the human TNBC tissues and cells, and noticed that PRKCD_pY313 showed a large difference between TNBC and normal tissues. PRKCD has been reported to promote survival and proliferation of transformed cells and tumor in a mouse models of cancer [29]. We investigated the role of PRKCD_Y313 in TNBC development in vivo and in vitro. We found that PRKCD_pY313 could inhibit apoptosis and promote migration and invasion of TNBC cells, while Dasatinib significantly attenuated these effects, which reminds us the potential applications in the patients with TNBC. A previous study stated that PRKCD is required in primary tumor stem cell lines, including breast, pancreas, prostate, and melanoma cells, and is sensitive to depletion of PRKCD or inhibitors [30]. Similarly, we observed that PRKCD knockdown inhibited the malignant biological behavior of TNBC cancer cells, which were closely associated with Src and p38 activity. Besides, Symonds et al. pointed to a role of PRKCD in promoting tumor growth, invasion, migration, and tumorigenesis [31], which is consistent with our findings. In addition, PRKCD was validated to promote the invasion and migration of colorectal cancer cells [32], control stress fiber formation in melanoma [33], participate in the invasion of thyroid cancer [34], and cause persistent KIT activation in colon cancer [35]. It is recognized that E-cadherin and N-cadherin expression and/or function variation is a hallmark of epithelial-mesenchymal transition (EMT) during tumorigenesis. Previous study confirmed that PRKCD was overexpressed in cervical cancer tissues, accompanied by the increased phosphorylation of E-cadherin at Thr790, which affected the EMT process [36]. Our study supported that PRKCD_pY313 affects the N-cadherin, Vimentin and ZO1 levels and promotes metastasis of TNBC, which might be accompanied by EMT process.

At present, the role of different levels of ROS in anti-cancer or cancer-promoting effects has been controversial. Cancer cells are characterized by increased production of ROS and altered oxidation-deoxidation environment compared to normal cells [37]. Continuous accumulation of ROS triggers oxidative stress, leading to excessive activation of signaling pathways that promote cell proliferation, survival, and metabolic adaptation to the tumor microenvironment [38]. However, excessive intracellular ROS induces apoptosis, and a large amount of ROS will cause cell necrosis. Mitochondria is the main site of ROS production [39]. The increase of ROS can lead to the opening of mitochondrial membrane permeability transport pore, which leads to the decrease of mitochondrial transmembrane potential and the release of cyto C, which in turn activates a series of caspase enzymes and induces apoptosis [39]. It has been suggested that Bcl-2 and Bcl-xL prevent the release of cyto c, thereby inhibiting programmed cell death, while bax and bak can promote this process [40], which is consistent with our results. Rottlerin (PKC inhibitor)-induced apoptosis is associated with cytochrome c release and activation of caspase 9 and caspase 3 cascades [41]. We noticed that PRKCD_pY313 increases Src activity and mitochondria membrane potential, and inhibit ROS production, accompanied by Bcl-2, Bax and cyto C variation. Conversely, free radical generation after ischemia-reperfusion injury mediates mitochondrial damage through PKCδ mitochondrial translocation [42–45]. Src is an oncogene that localizes to multiple subcellular regions and participates in several important signaling pathways of proliferation, differentiation, invasion and metastasis, and angiogenesis [46]. Intra-mitochondrial Src (mtSrc) is contained in different types of cancer cells, including breast cancer cells [47]. The mtSrc signaling pathway is involved in different mitochondrial functions ranging from ATP and ROS production to apoptosis [48, 49]. In mitochondria, Src targets a variety of proteins to regulate the activity of organelles. For example, it targets the mitochondrial single-stranded DNA-binding protein, which is a regulator of mitochondrial DNA replication. Src is positively associated with increased invasive and metastatic capacity [50] and reduced patient survival [51]. In contrast, inhibition of Src activity reduces the malignant biological behavior of tumor cells [52–54]. Since tumor cells are more sensitive to ROS than normal cells, ROS can selectively kill tumor cells [55]. Based on forward technology [56], the detailed mechanism and site of PRKCD and Src need to be further studied. The application of pro-oxidant drugs and kinase inhibitors in tumor treatment still needs to be further studied, and the specific mechanism of its anti-tumor effect also needs to be further explored.

Furthermore, PRKCD_pY313 overexpression showed the opposite effects to low PRKCD_pY313 level, mirroring that pY313 is essential for PRKCD activity regulation in TNBC progression. Based on the literature review, we speculated that the phosphorylation of tyrosine residue 313 of PRKCD may bind to the C2 region of PRKCD, thereby causing a conformational change of PRKCD and exposing the corresponding C4 catalytic region, and thus activating the kinase to perform its function [22]. In addition, Y313 site is in the hinge region of PRKCD, which can be cleaved by caspase to release nuclear targeting signal [21]. However, we found that cleaved caspase 3 or PRKCD levels increased with Y313F or decreased with PRKCD_pY313. Further studies are needed to determine the effect of Y313 on PRKCD conformation and caspase cleavage function.

Taken together, we demonstrate that PRKCD_pY313 is important for PRKCD kinase activity and plays an important role in TNBC progression through activating Src, making it a valuable target, which provides theoretical support for the application of Dasatinib in the treatment of TNBC. Further studies are needed to develop PRKCD_Y313 selective kinase inhibitors and effective kinase inhibitors and their combination regimens.

### Supplementary Information

**Additional file 1: Table S1.** PRKCD knockout-sgRNA sequences. **Table S2.** PRKCD knockdown shRNA primers. **Table S3.** PRKCD overexpression and Y313F mutation amplification primers. **Table S4.** Realtime RT-PCR primers. **Table S5.** The primary antibodies used in western blot analysis. **Fig. S1.** Functional enrichment analysis of significant serine/threonine peptides in breast cancer tissues. (A) Barplot of Gene Ontology analysis of significant serine/threonine peptides peptides related genes. (B) Barplot of top 10 KEGG pathways of significant serine/threonine peptides peptides related genes. KEGG, Kyoto Encyclopedia of Genes and Genomes. **Fig. S2.** Personalized kinase activity map of patient with Luminal A subtype of breast cancer. Each protein frame is divided into four small squares, each of which represents one pY, pS or pT site. The color represents the log_10_(Ca/N) value, red represents the upregulation of this site, and the darker the color, the higher the upregulation ratio. Blue indicates downregulation, darker color indicates higher downregulation, and gray indicates no such site. The color of the border of each protein bar represents the sum of the log_10_(Ca/N) values of all sites of the protein. The thickness of the lines between the proteins indicates a test score ranging from 1 to 10, and the color of the lines ranging from gray to red indicates a test score ranging from 0 to 1. Ca/N, ratio of expression levels in breast cancer tumors to that in normal breast tissue. **Fig. S3.** Personalized kinase activity map of patient with Luminal B subtype of breast cancer. Each protein frame is divided into four small squares, each of which represents one pY, pS or pT site. The color represents the log_10_(Ca/N) value, red represents the upregulation of this site, and the darker the color, the higher the upregulation ratio. Blue indicates downregulation, darker color indicates higher downregulation, and gray indicates no such site. The color of the border of each protein bar represents the sum of the log_10_(Ca/N) values of all sites of the protein. The thickness of the lines between the proteins indicates a test score ranging from 1 to 10, and the color of the lines ranging from gray to red indicates a test score ranging from 0 to 1. Ca/N, ratio of expression levels in breast cancer tumors to that in normal breast tissue. **Fig. S4.** Personalized kinase activity map of patient with HER2 positive subtype of breast cancer. Each protein frame is divided into four small squares, each of which represents one pY, pS or pT site. The color represents the log_10_(Ca/N) value, red represents the upregulation of this site, and the darker the color, the higher the upregulation ratio. Blue indicates downregulation, darker color indicates higher downregulation, and gray indicates no such site. The color of the border of each protein bar represents the sum of the log_10_(Ca/N) values of all sites of the protein. The thickness of the lines between the proteins indicates a test score ranging from 1 to 10, and the color of the lines ranging from gray to red indicates a test score ranging from 0 to 1. Ca/N, ratio of expression levels in breast cancer tumors to that in normal breast tissue. **Fig. S5.** Top five Gene Ontology-biological process pathways of significant pY/pS/pT peptides related genes in breast tissues. **Fig. S6.** Top five Gene Ontology-cellular component pathways of significant pY/pS/pT peptides related genes in breast tissues. **Fig. S7.** Top five Gene Ontology-molecular function pathways of significant pY/pS/pT peptides related genes in breast tissues. **Fig. S8.** Phosphorylated tyrosine/serine/threonine related genes in pathway of proteoglycans in cancer. The KEGG pathway enrichment results of genes from our mass spectrum data in pathway of proteoglycans in cancer. All colored rectangles represent genes corresponding to phosphorylated polypeptide proteins enriched in our mass spectrometry analysis. Red represents increased phosphorylation of this protein in tumor tissue, and green represents decreased phosphorylation of this protein in tumor tissue. This pathway includes hyaluronan, chondroitin sulfate/dermatan sulfate proteoglycan, keratan sulfate proteoglycan, and heparan sulfate proteoglycans. **Fig. S9.** Phosphorylated tyrosine/serine/threonine related genes in pathway of focal adhesion. The KEGG pathway enrichment results of genes from our mass spectrum data in pathway of focal adhesion. All colored rectangles represent genes corresponding to phosphorylated polypeptide proteins enriched in our mass spectrometry analysis. Red represents increased phosphorylation of this protein in tumor tissue, and green represents decreased phosphorylation of this protein in tumor tissue. This pathway includes ECM-receptor interaction, cytokine-cytokine receptor interaction, which could affect the cell motility, cell proliferation and cell survival. **Fig. S10.** Phosphorylated tyrosine/serine/threonine related genes in ErbB signaling pathway. The KEGG pathway enrichment results of genes from our mass spectrum data in ErbB signaling pathway. All colored rectangles represent genes corresponding to phosphorylated polypeptide proteins enriched in our mass spectrometry analysis. Red represents increased phosphorylation of this protein in tumor tissue, and green represents decreased phosphorylation of this protein in tumor tissue. This pathway includes ERBB1, ERBB2, ERBB3, and ERBB4, which could affect the proliferation of pancreatic cancer and non-small cell lung cancer, the differentiation of glioma and endometrial cancer. **Fig. S11.** Phosphorylated tyrosine/serine/threonine related genes in pathway of tight junction. The KEGG pathway enrichment results of genes from our mass spectrum data in pathway of tight junction. All colored rectangles represent genes corresponding to phosphorylated polypeptide proteins enriched in our mass spectrometry analysis. Red represents increased phosphorylation of this protein in tumor tissue, and green represents decreased phosphorylation of this protein in tumor tissue. This pathway includes claudin, occluding, JAM, bves, which could affect the cell polarity, cell proliferation, cell survival, cell differentiation, cell migration. **Fig. S12.** Phosphorylated tyrosine/serine/threonine related genes in pathway of adherens junction. The KEGG pathway enrichment results of genes from our mass spectrum data in pathway of adherens junction. All colored rectangles represent genes corresponding to phosphorylated polypeptide proteins enriched in our mass spectrometry analysis. Red represents increased phosphorylation of this protein in tumor tissue, and green represents decreased phosphorylation of this protein in tumor tissue. This pathway includes nectin (strong adhesion), cadherin (weak adhesion) et al. **Fig. S13.** Phosphorylated tyrosine/serine/threonine related genes in MAPK signaling pathway. The KEGG pathway enrichment results of genes from our mass spectrum data in MAPK signaling pathway. All colored rectangles represent genes corresponding to phosphorylated polypeptide proteins enriched in our mass spectrometry analysis. Red represents increased phosphorylation of this protein in tumor tissue, and green represents decreased phosphorylation of this protein in tumor tissue. This pathway includes classical MAP kinase pathway, JNK and p38 MAP kinase pathway, and ERK5 pathway, which could affect the cell proliferation, cell cycle, cell differentiation, and cell inflammation. **Fig. S14.** Survival analysis of patients with breast cancer in different PRKCD level. (A) Kaplan-Meier plot of disease specific survival of patients with breast cancer in different PRKCD level (DATASET: GSE3494-GPL96). (B) Kaplan-Meier plot of distant metastasis free survival of patients with breast cancer in different PRKCD level (DATASET: GSE9195). HR, Hazard Ratio. **Fig. S15.** PRKCD_pY313 promotes malignant biological behaviors of triple-negative breast cancer cells. (A) The knockdown and knockout rate of PRKCD and the PRKCD_pY313 level in MDA-MB-231 stable transfected cell lines. (B) Apoptosis rate of stable transfected MDA-MB-231 and BT549 cell lines with different PRKCD_pY313 level, detected by flow cytometry. (C) The colony formation rate of MDA-MB-231 and BT549 cell lines with different PRKCD_pY313 level. (D) The wound healing assay of stable MDA-MB-231 and BT549 cell lines with different PRKCD_pY313 level. (E) Invasion assay of stable transfected MDA-MB-231 and BT549 cell lines with different PRKCD_pY313 level. OE, overexpression; KD, knockdown. KO, knockout. Mean ± SD, *n* = 3 per group, * *P* < 0.05, ** *P* < 0.01, *** *P* < 0.001, **** *P* < 0.0001. **Fig. S16.** Expression levels of proteins involved in apoptosis and invasion and metastasis of MDA-MB-231 and BT549 cell lines. (A) Quantification of Fig. 5F, apoptotic proteins in MDA-MB-231 cell lines with overexpressed PRKCD_pY313 or PRKCD_Y313F. (B) Quantification of Fig. 5F, apoptotic proteins in MDA-MB-231 cell lines with low PRKCD_pY313 level. (C) Quantification of Fig. 5F, apoptotic proteins in BT549 cell lines with overexpressed PRKCD_pY313 or PRKCD_Y313F. (D) Quantification of Fig. 5F, proteins that mark metastatic capacity in MDA-MB-231 cell lines with overexpressed PRKCD_pY313 or PRKCD_Y313F level. (E) Quantification of Fig. 5F, Proteins that mark metastatic capacity in MDA-MB-231 cell lines with low PRKCD_pY313 level. (F) Quantification of Fig. 5F, Proteins that mark metastatic capacity in BT549 cell lines with overexpressed PRKCD_pY313 or PRKCD_Y313F level. Mean ± SD, *n* = 3 per group, * *P* < 0.05, ** *P* < 0.01, *** *P* < 0.001, **** *P* < 0.0001. **Fig. S17.** Proteins and phosphorylated levels of PRKCD-related kinases in MDA-MB-231 and BT549 cell lines with different PRKCD_pY313 level. (A) Quantification of Fig. 7A. (B) Quantification of Fig. 7B.(C) Quantification of Fig. 7C. Mean ± SD, *n* = 3 per group, * *P* < 0.05, ** *P* < 0.01, *** *P* < 0.001, **** *P* < 0.0001. **Fig. S18.** Proteins and phosphorylated levels of PRKCD-related kinases in MDA-MB-231 and BT549 cell lines with dasatinib treatment. (A) Quantification of Src_pY419/Src level in MDA-MB-231 cell lines in Fig. 7D. (B) Quantification of kinases’ activity in MDA-MB-231 cell lines in Fig. 7D. (C) Quantification of Src_pY419/Src level in BT549 cell lines in Fig. 7E. (D) Quantification of kinases’ activity in BT549 cell lines in Fig. 7E. Mean ± SD, *n* = 3 per group, * *P* < 0.05, ** *P* < 0.01, *** *P* < 0.001, **** *P* < 0.0001. **Fig. S19.** The MTT assay of stable MDA-MB-231 and BT549 cells treated with dasatinib. (A) The cell relative viability of MDA-MB-231 cell with dasatinib treatment and IC50 (Half maximal inhibitory concentration). (B) The cell relative viability of BT549 cell with dasatinib treatment and IC50. (C) The cell relative viability of MDA-MB-231 cell with 0.01 μM dasatinib treatment. (D) The cell relative viability of BT549 cell with 0.01 μM dasatinib treatment. (E) The cell relative viability of MDA-MB-231 cell with 0.025 μM dasatinib treatment. (F) The cell relative viability of BT549 cell with 0.025 μM dasatinib treatment. (G) The cell relative viability of MDA-MB-231 cell with 0.05 μM dasatinib treatment. (H) The cell relative viability of BT549 cell with 0.05 μM dasatinib treatment. (I) The cell relative viability of MDA-MB-231 cell with 0.1 μM dasatinib treatment. (J) The cell relative viability of BT549 cell with 0.1 μM dasatinib treatment. (K) The cell relative viability of MDA-MB-231 cell with 0.2 μM dasatinib treatment. (L) The cell relative viability of BT549 cell with 0.2 μM dasatinib treatment. Mean ± SD, *n* = 3 per group, * *P* < 0.05, ** *P* < 0.01, *** *P* < 0.001, **** *P* < 0.0001.

## Data Availability

The datasets supporting the conclusions of this article are included within the article and its additional files.

## Abbreviations

BP: biological process
CC: cellular component
Ca/N: ratio of expression levels in breast cancer tumors to that in normal breast tissue
CPTAC: Clinical Proteomic Tumor Analysis Consortium
DAG: diacylglycerol
DHE: Dihydroethidium
ER: Estrogen receptor
EMT: epithelial-mesenchymal transition
PR: progesterone receptor
HER2: human epidermal growth factor receptor 2
FC: fold change
FBS: fetal bovine serum
FDR: false discovery rate
GO: gene ontology
KEGG: Kyoto Encyclopedia of Genes and Genomes
MF: molecular function
MAPK: mitogen-activated protein kinases
MS: mass spectrometry
MMP: mitochondrial membrane potential
N stage: nodal stage
IHC: immunohistochemistry
pY: phosphorylated tyrosine
pS/pT: phosphorylated serine or threonine
pY/pS/pT: phosphorylated tyrosine or serine or threonine
PTM: post-translational modification
PKC: Protein kinase C
PMA: Phorbol 12-myristate 13-acetate
PRM: Parallel reaction monitoring
T stage: tumor stage
TKs: tyrosine kinases
TNBC: triple negative breast cancer
TCGA: The Cancer Genome Atlas Program
shRNA: short hairpin RNA
SD: standard deviation
OE: overexpression
