# Supplementary material for: Superbinder based phosphoproteomic landscape revealed PRKCD_pY313 mediates the activation of Src and p38 MAPK to promote TNBC progression
Source: Cell Commun Signal. 2024 Feb 12;22:115. doi: 10.1186/s12964-024-01487-z (PMC10860301; doi:10.1186/s12964-024-01487-z)

**Superbinder Based Phosphoproteomic Landscape Revealed PRKCD_pY313 Mediates the Activation of Src and p38 MAPK to Promote TNBC Progression**

Yujiao Deng^1,2,†^, Zhanwu Hou^3,^^†^, Yizhen Li^2,†^, Ming Yi^4^, Ying Wu^2^, Yi Zheng^2^, Fei Yang^3^, Guansheng Zhong^4^, Qian Hao^2^, Zhen Zhai^2^, Meng Wang^2^, Xiaobin Ma^2^, Huafeng Kang^2^, Fanpu Ji^5^, Chenfang Dong^6,7^^,*^, Huadong Liu ^3,*^, and Zhijun Dai^2,4,*^

[Supplemental Files 1](#_Toc155600023)

[Table S1 PRKCD knockout-sgRNA sequences 1](#_Toc155600024)

[Table S2 PRKCD knockdown shRNA primers 1](#_Toc155600025)

[Table S3 PRKCD overexpression and Y313F mutation amplification primers 1](#_Toc155600026)

[Table S4 Realtime RT-PCR primers 2](#_Toc155600027)

[Table S5 Primary antibodies used in western blot analysis 2](#_Toc155600028)

[Figure S1 Functional enrichment analysis of significant serine/threonine peptides in breast cancer tissues 4](#_Toc155600029)

[Figure S2 Personalized kinase activity map of patient with Luminal A subtype of breast cancer 5](#_Toc155600030)

[Figure S3 Personalized kinase activity map of patient with Luminal B subtype of breast cancer 6](#_Toc155600031)

[Figure S4 Personalized kinase activity map of patient with Her-2 positive subtype of breast cancer 7](#_Toc155600032)

[Figure S5 Top five Gene Ontology-biological process pathways of significant pY/pS/pT peptides related genes in breast tissues 8](#_Toc155600033)

[Figure S6 Top five Gene Ontology-cellular component pathways of significant pY/pS/pT peptides related genes in breast tissues 9](#_Toc155600034)

[Figure S7 Top five Gene Ontology-molecular function pathways of significant pY/pS/pT peptides related genes in breast tissues 10](#_Toc155600035)

[Figure S8 Phosphorylated tyrosine/serine/threonine related genes in pathway of proteoglycans in cancer 11](#_Toc155600036)

[Figure S9 Phosphorylated tyrosine/serine/threonine related genes in pathway of focal adhesion 11](#_Toc155600037)

[Figure S10 Phosphorylated tyrosine/serine/threonine related genes in ErbB signaling pathway 13](#_Toc155600038)

[Figure S11 Phosphorylated tyrosine/serine/threonine related genes in pathway of tight junction 14](#_Toc155600039)

[Figure S12 Phosphorylated tyrosine/serine/threonine related genes in pathway of adherens junction 15](#_Toc155600040)

[Figure S13 Phosphorylated tyrosine/serine/threonine related genes in MAPK signaling pathway 16](#_Toc155600041)

[Figure S14 Survival analysis of patients with breast cancer in different PRKCD level 17](#_Toc155600042)

[Figure S15 PRKCD_pY313 promotes malignant biological behaviors of triple-negative breast cancer cells 18](#_Toc155600043)

[Figure S16 Expression levels of proteins involved in apoptosis and invasion and metastasis of MDA-MB-231 and BT549 cell lines 19](#_Toc155600044)

[Figure S17 Proteins and phosphorylated levels of PRKCD-related kinases in MDA-MB-231 and BT549 cell lines with different PRKCD_pY313 level 20](#_Toc155600045)

[Figure S18 Proteins and phosphorylated levels of PRKCD-related kinases in MDA-MB-231 and BT549 cell lines with dasatinib treatment 21](#_Toc155600046)

[Figure S19 The MTT assay of stable MDA-MB-231 and BT549 cells treated with dasatinib 22](#_Toc155600047)

# Supplemental Files

## Table S1 PRKCD knockout-sgRNA sequences

| Name | Sequences |
| --- | --- |
| Lentiv2_PRKCD_gRNA_F | 5’-CACCGtgcagagcgtgggaaaacac -3’ |
| Lentiv2_PRKCD_gRNA_R | 5’-AAACgtgttttcccacgctctgcaC -3’ |

## Table S2 PRKCD knockdown shRNA primers

| Name | Sequences |
| --- | --- |
| Nontarget | Forward: 5’-CCGGCAGAGCCTGTTGGGATATATCCTCGAGGATATATCCCAACAGGCTCTGTTTTTG -3’ |
|  | Reverse: 5’-AATTCAAAAACAGAGCCTGTTGGGATATATCCTCGAGGATATATCCCAACAGGCTCTG -3’ |
| PRKCD-KD1 | Forward: 5’-CCGGGGCCGCTTTGAACTCTACCGTCTCGAGACGGTAGAGTTCAAAGCGGCCTTTTTG -3’ |
|  | Reverse: 5’-AATTCAAAAAGGCCGCTTTGAACTCTACCGTCTCGAGACGGTAGAGTTCAAAGCGGCC -3’ |
| PRKCD-KD2 | Forward: 5’-CCGGCAGAGCCTGTTGGGATATATCCTCGAGGATATATCCCAACAGGCTCTGTTTTTG -3’ |
|  | Reverse: 5’-AATTCAAAAACAGAGCCTGTTGGGATATATCCTCGAGGATATATCCCAACAGGCTCTG -3’ |

## Table S3 PRKCD overexpression and Y313F mutation amplification primers

| Name | Primer sequences |
| --- | --- |
| OE-PRKCD-NheⅠ-F | 5’-CTAGCTAGCAAAGTTGGCGACTACAAAGACGAT-3’ |
| OE-PRKCD-NotⅠ-R | 5’-CATTTGCGGCCGCTCAATCTTCCAGGAGGTGCT-3’ |
| PRKCD-a938t-NheⅠ-F | 5’-CCTCAGAGCCTGTTGGGATATTTCAGGGTTTCG-3’ |
| PRKCD-a938t-R | 5’-CGAAACCCTGAAATATCCCAACAGGCTCTGAGG-3’ |
| PRKCD-A938T-F2 | 5’-TGGGATATTTCAGGGTTTCGAGAAGAAGACCGGAGT-3’ |
| PRKCD-A938T NotⅠ-R2 | 5’-GAAACCCTGAAATATCCCAACAGGCTCTGAGGAGGCT-3’ |
| PRKCD-T180C- NheⅠ-F | 5’-TGAGTGGAAGTCGACGTTCGACGCCCACATCTAT-3’ |
| PRKCD-T180C-R | 5’-ATAGATGTGGGCGTCGAACGTCGACTTCCACTCA-3’ |
| PRKCD-T180C-F2 | 5’-GACGTTCGACGCCCACATCTATGAGGGGCGCGTCAT-3’ |
| PRKCD-T180C-NotⅠ-R2 | 5’-AGATGTGGGCGTCGAACGTCGACTTCCACTCAGGAT-3’ |

## Table S4 Realtime RT-PCR primers

| Gene | Primer sequences |
| --- | --- |
| PRKCD | Forward：5’-GCTGACACTTGCCGCAGAGAAT-3’  Reverse：5’-GCCTTTGTCCTGGATGTGGTAC-3’ |
| β-actin | Forward：5’-CCACACCTTCTACAATGAGC-3’  Reverse：5’-GGTCTCAAACATGATCTGGG-3’ |

## Table S5 Primary antibodies used in western blot analysis

| Antibodies | Dilution | Source |
| --- | --- | --- |
| GAPDH | 1:5000 | Cell signal technology |
| p-PRKCD(Y313) | 1:1000 | Cell signal technology |
| PRKCD | 1:2000 | Abcam |
| mTOR | 1:500 | Santa Cruz Biotechnology |
| p-mTOR (S2448) | 1:500 | Santa Cruz Biotechnology |
| Akt | 1:500 | Santa Cruz Biotechnology |
| p-Akt (S473) | 1:500 | Santa Cruz Biotechnology |
| GSK3β | 1:500 | Santa Cruz Biotechnology |
| GSK3β_pS9 | 1:500 | Santa Cruz Biotechnology |
| β-catenin | 1:1000 | Cell signal technology |
| Vimentin | 1:500 | Santa Cruz Biotechnology |
| N-cadherin | 1:500 | Santa Cruz Biotechnology |
| Src | 1:1000 | Cell signal technology |
| p-Src(S416) | 1:1000 | Cell signal technology |
| ERK1/2 | 1:1000 | Cell signal technology |
| p-ERK1/2(T202/Y204) | 1:1000 | Cell signal technology |
| P38 | 1:500 | Santa Cruz Biotechnology |
| p-P38(T180/Y182) | 1:500 | Santa Cruz Biotechnology |
| cyto C | 1:1000 | Cell signaling technology |
| Bcl2 | 1:500 | Santa Cruz Biotechnology |
| Bax | 1:500 | Santa Cruz Biotechnology |

## Figure S1 Functional enrichment analysis of significant serine/threonine peptides in breast cancer tissues

(A) Barplot of Gene Ontology analysis of significant serine/threonine peptides peptides related genes. (B) Barplot of top 10 KEGG pathways of significant serine/threonine peptides peptides related genes. KEGG, Kyoto Encyclopedia of Genes and Genomes.


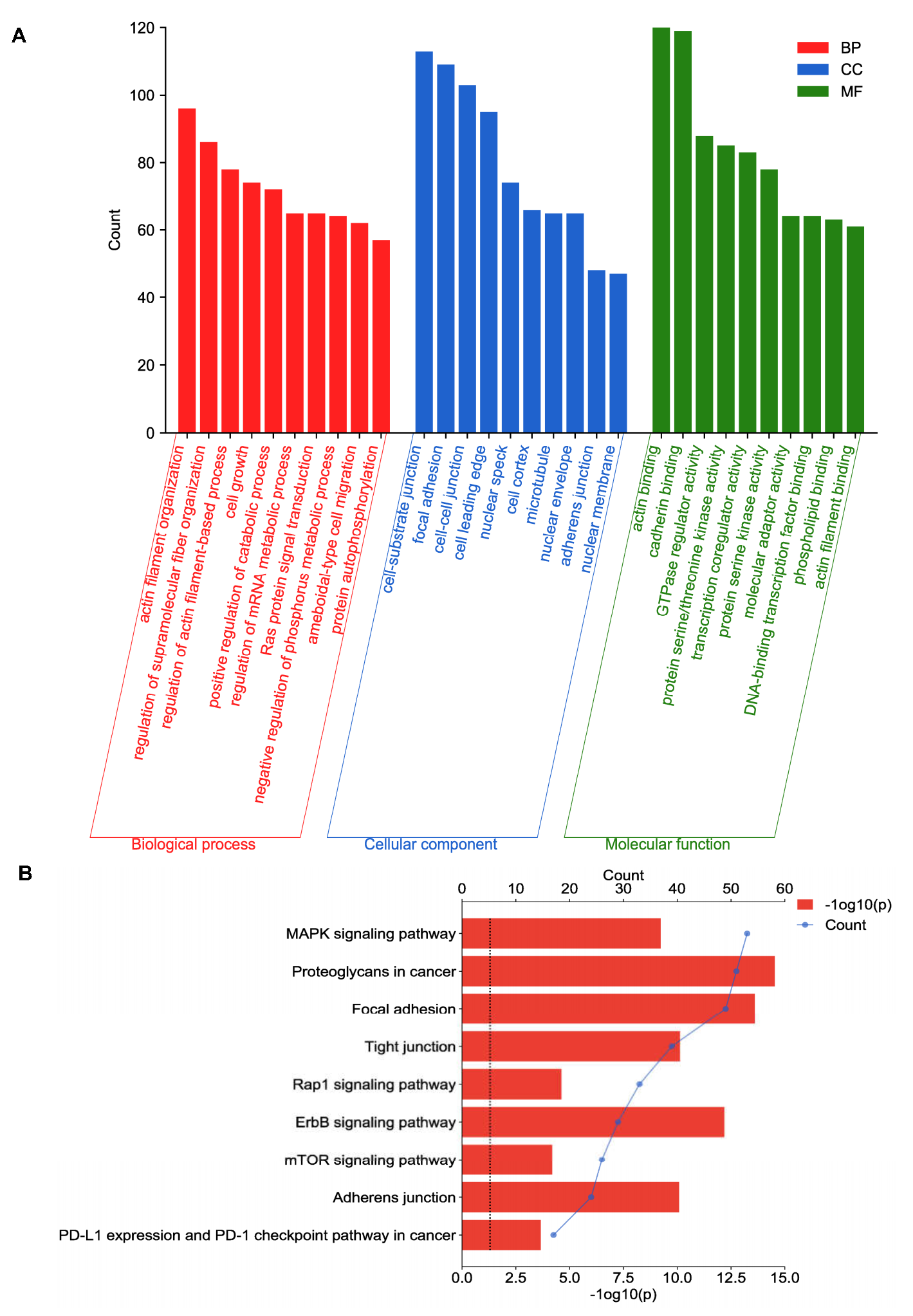


## Figure S2 Personalized kinase activity map of patient with Luminal A subtype of breast cancer

Each protein frame is divided into four small squares, each of which represents one pY, pS or pT site. The color represents the log_10_(Ca/N) value, red represents the upregulation of this site, and the darker the color, the higher the upregulation ratio. Blue indicates downregulation, darker color indicates higher downregulation, and gray indicates no such site. The color of the border of each protein bar represents the sum of the log_10_(Ca/N) values of all sites of the protein. The thickness of the lines between the proteins indicates a test score ranging from 1 to 10, and the color of the lines ranging from gray to red indicates a test score ranging from 0 to 1. Ca/N, ratio of expression levels in breast cancer tumors to that in normal breast tissue.


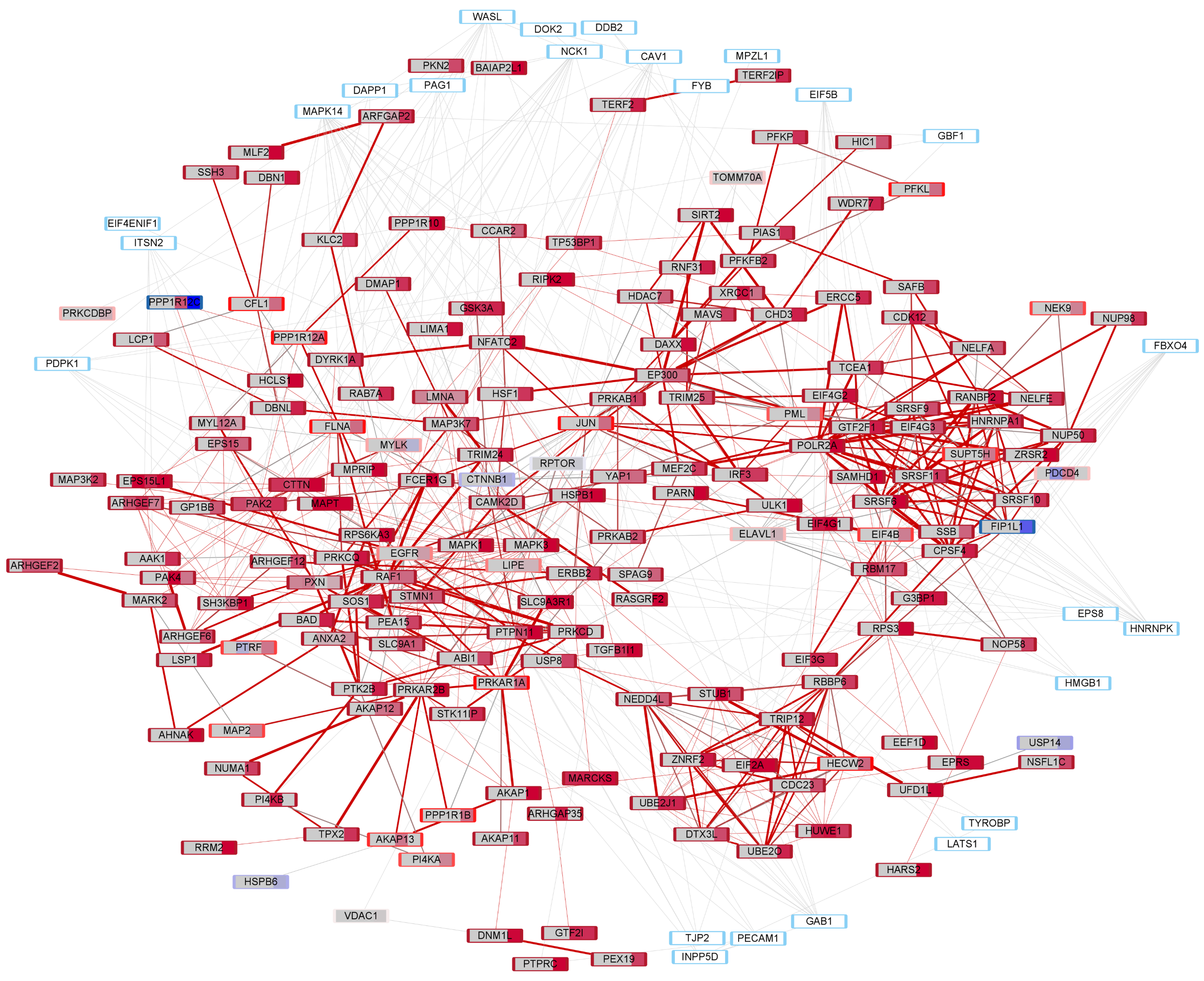


## Figure S3 Personalized kinase activity map of patient with Luminal B subtype of breast cancer

Each protein frame is divided into four small squares, each of which represents one pY, pS or pT site. The color represents the log_10_(Ca/N) value, red represents the upregulation of this site, and the darker the color, the higher the upregulation ratio. Blue indicates downregulation, darker color indicates higher downregulation, and gray indicates no such site. The color of the border of each protein bar represents the sum of the log_10_(Ca/N) values of all sites of the protein. The thickness of the lines between the proteins indicates a test score ranging from 1 to 10, and the color of the lines ranging from gray to red indicates a test score ranging from 0 to 1. Ca/N, ratio of expression levels in breast cancer tumors to that in normal breast tissue.


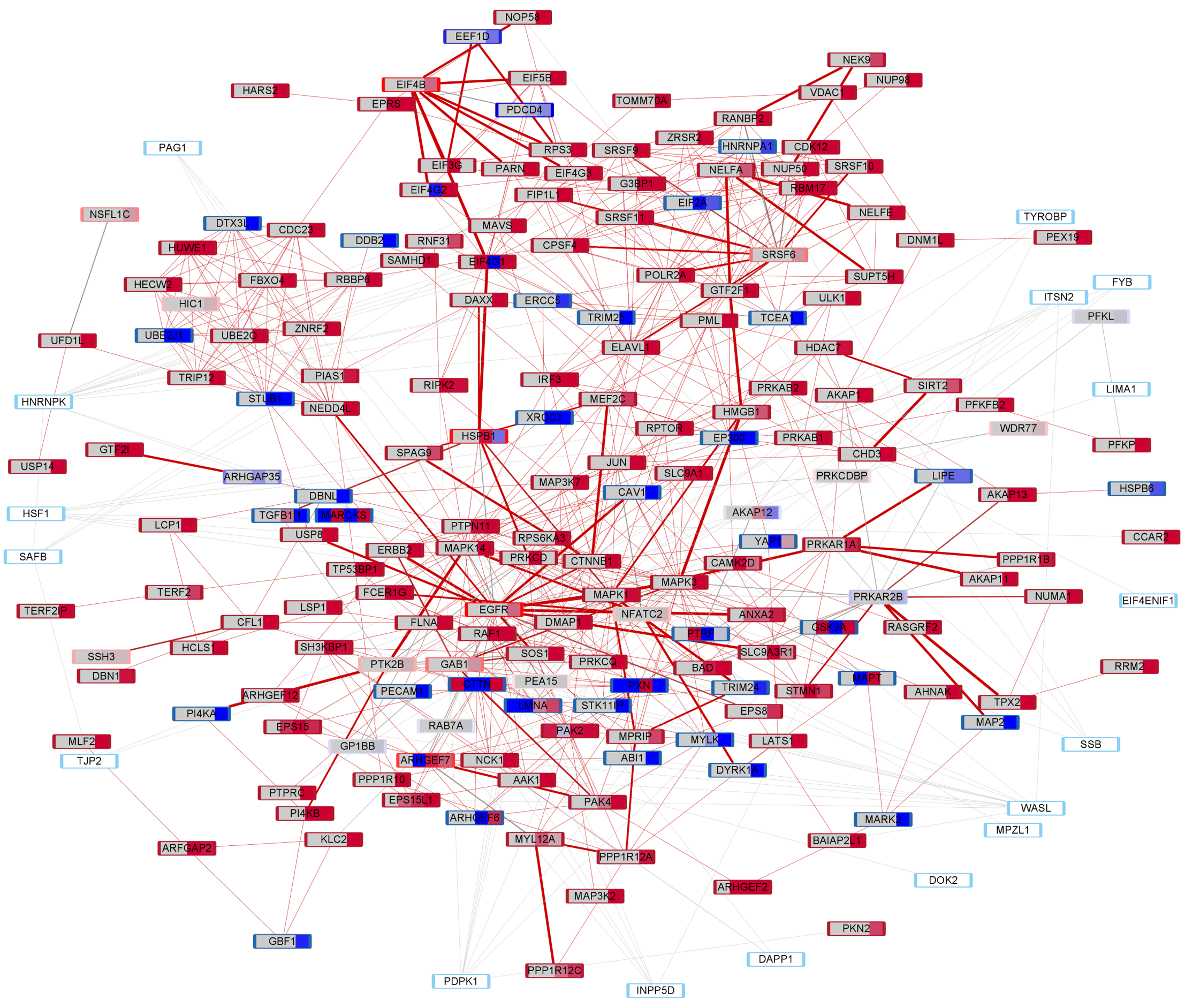


## Figure S4 Personalized kinase activity map of patient with Her-2 positive subtype of breast cancer

Each protein frame is divided into four small squares, each of which represents one pY, pS or pT site. The color represents the log_10_(Ca/N) value, red represents the upregulation of this site, and the darker the color, the higher the upregulation ratio. Blue indicates downregulation, darker color indicates higher downregulation, and gray indicates no such site. The color of the border of each protein bar represents the sum of the log_10_(Ca/N) values of all sites of the protein. The thickness of the lines between the proteins indicates a test score ranging from 1 to 10, and the color of the lines ranging from gray to red indicates a test score ranging from 0 to 1. Ca/N, ratio of expression levels in breast cancer tumors to that in normal breast tissue.


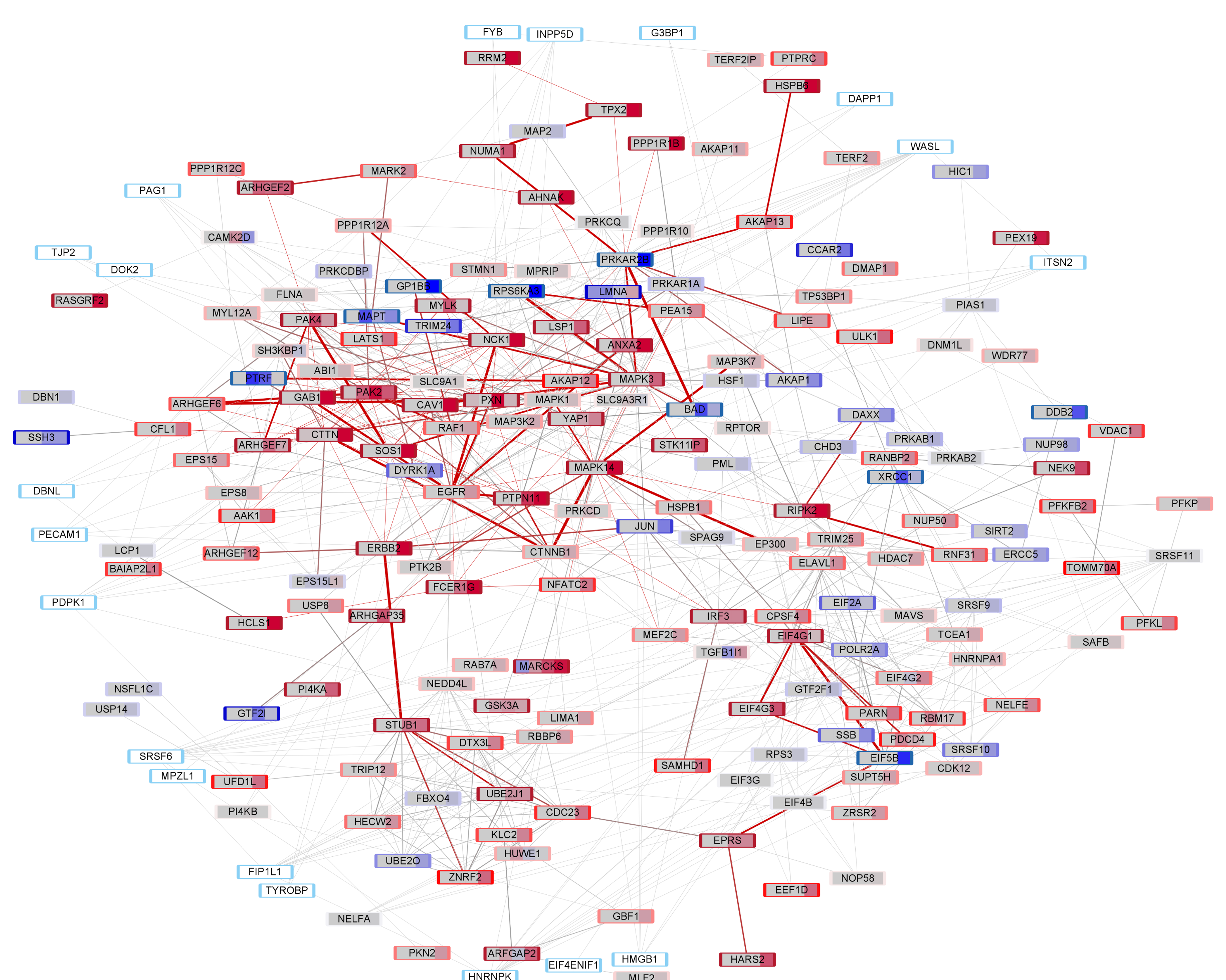


## Figure S5 Top five Gene Ontology-biological process pathways of significant pY/pS/pT peptides related genes in breast tissues


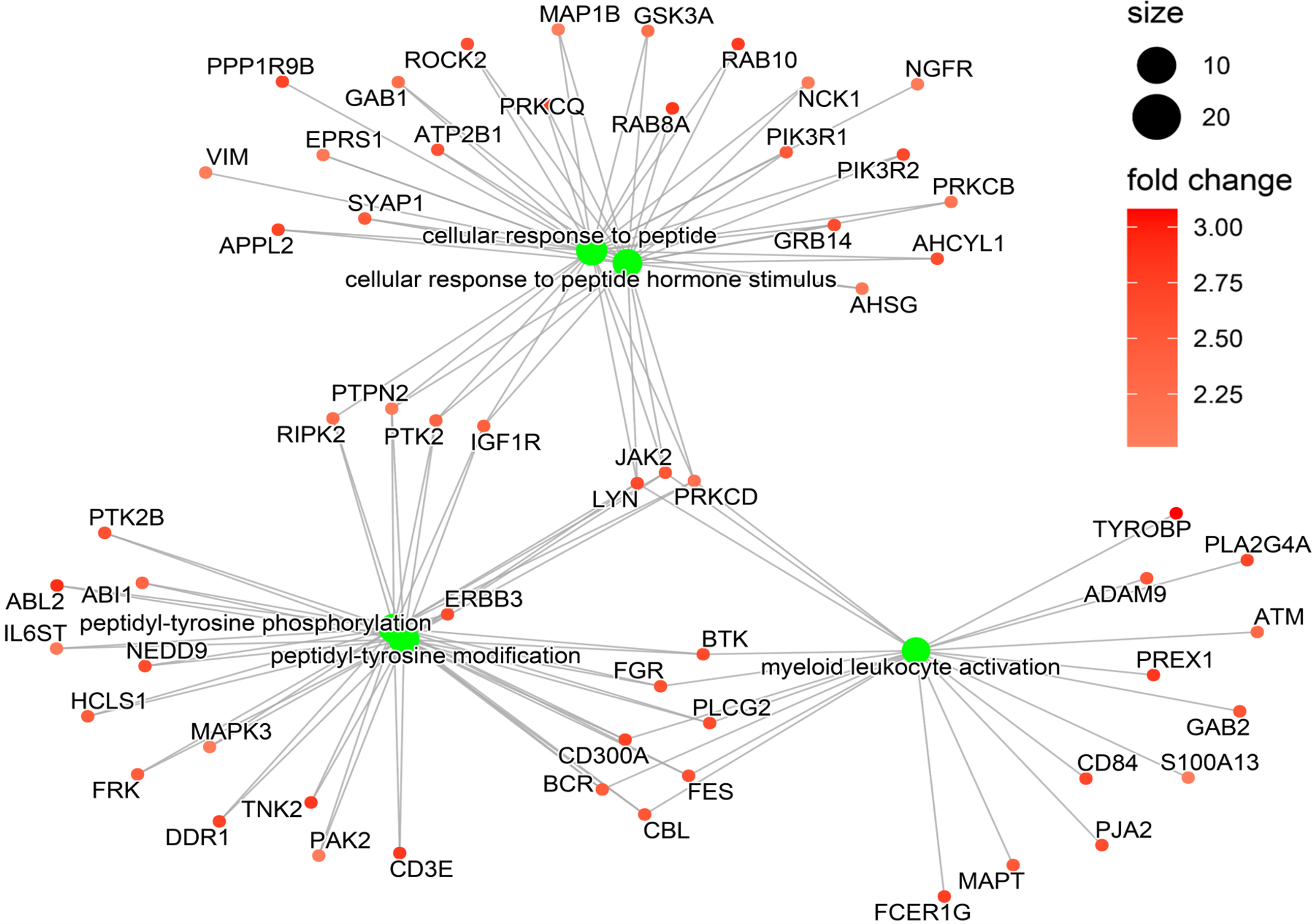


## Figure S6 Top five Gene Ontology-cellular component pathways of significant pY/pS/pT peptides related genes in breast tissues


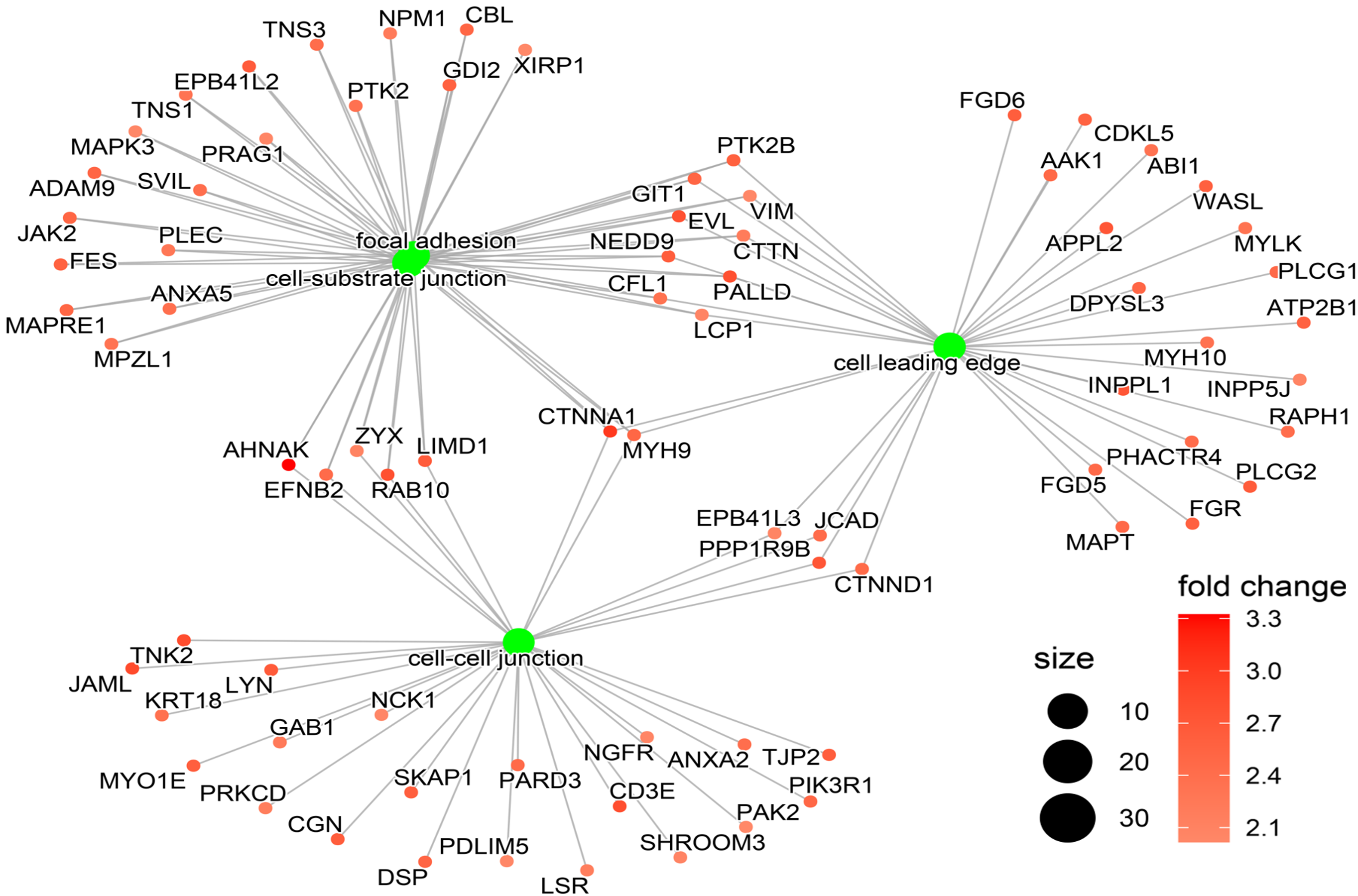


## Figure S7 Top five Gene Ontology-molecular function pathways of significant pY/pS/pT peptides related genes in breast tissues


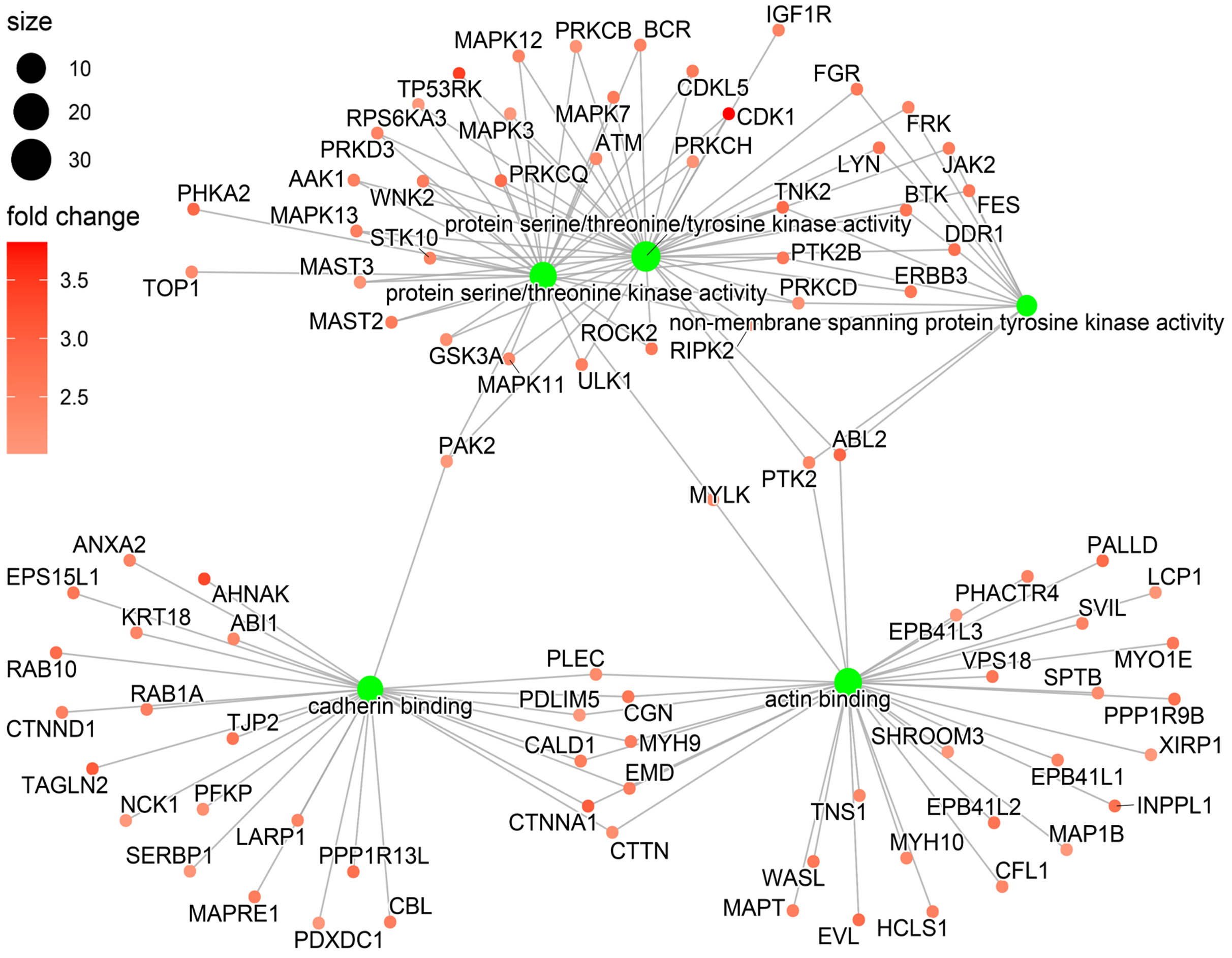


## Figure S8 Phosphorylated tyrosine/serine/threonine related genes in pathway of proteoglycans in cancer

The KEGG pathway enrichment results of genes from our mass spectrum data in pathway of proteoglycans in cancer. All colored rectangles represent genes corresponding to phosphorylated polypeptide proteins enriched in our mass spectrometry analysis. Red represents increased phosphorylation of this protein in tumor tissue, and green represents decreased phosphorylation of this protein in tumor tissue. This pathway includes hyaluronan, chondroitin sulfate/dermatan sulfate proteoglycan, keratan sulfate proteoglycan, and heparan sulfate proteoglycans.


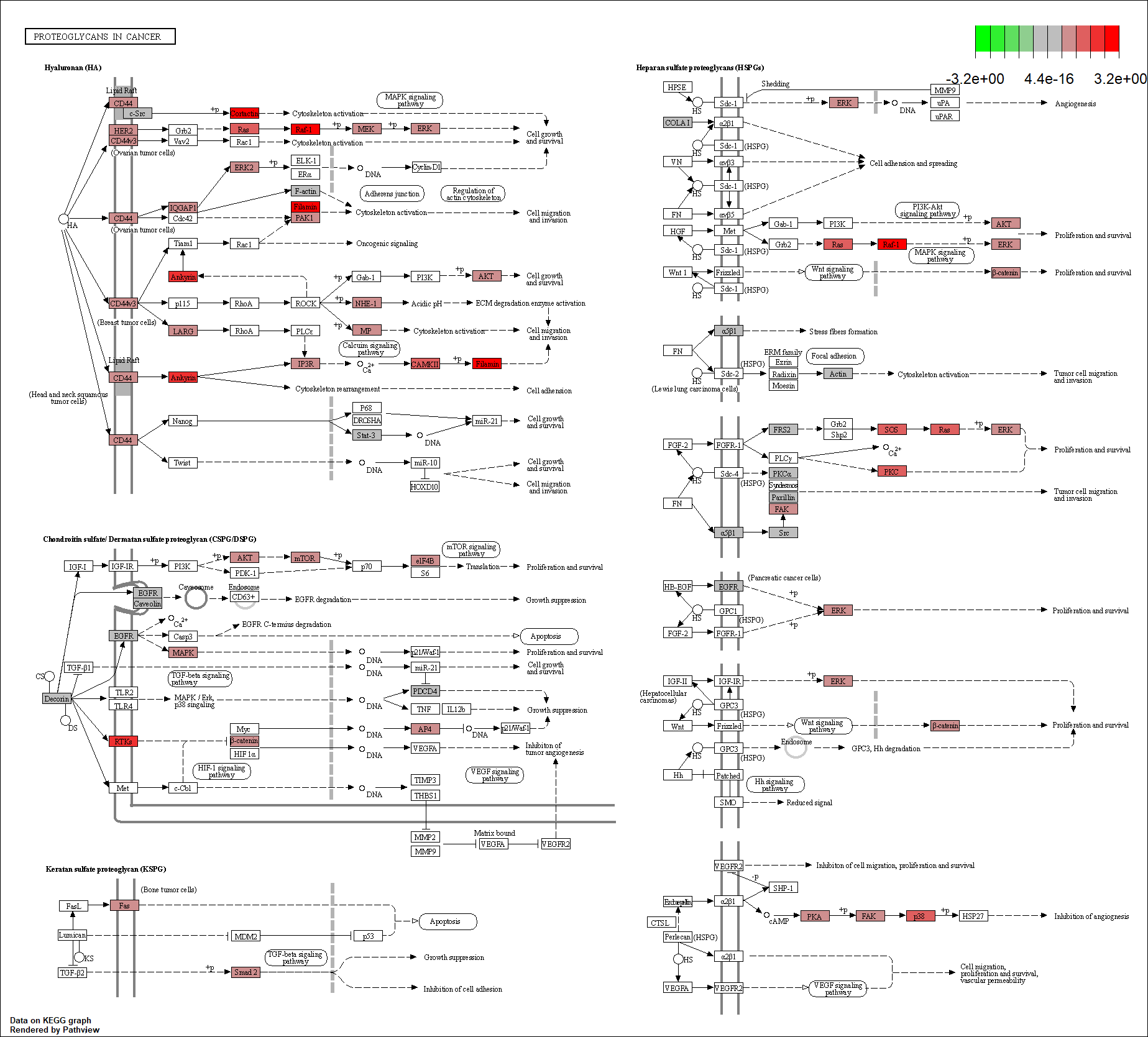


## Figure S9 Phosphorylated tyrosine/serine/threonine related genes in pathway of focal adhesion

The KEGG pathway enrichment results of genes from our mass spectrum data in pathway of focal adhesion. All colored rectangles represent genes corresponding to phosphorylated polypeptide proteins enriched in our mass spectrometry analysis. Red represents increased phosphorylation of this protein in tumor tissue, and green represents decreased phosphorylation of this protein in tumor tissue. This pathway includes ECM-receptor interaction, cytokine-cytokine receptor interaction, which could affect the cell motility, cell proliferation and cell survival.


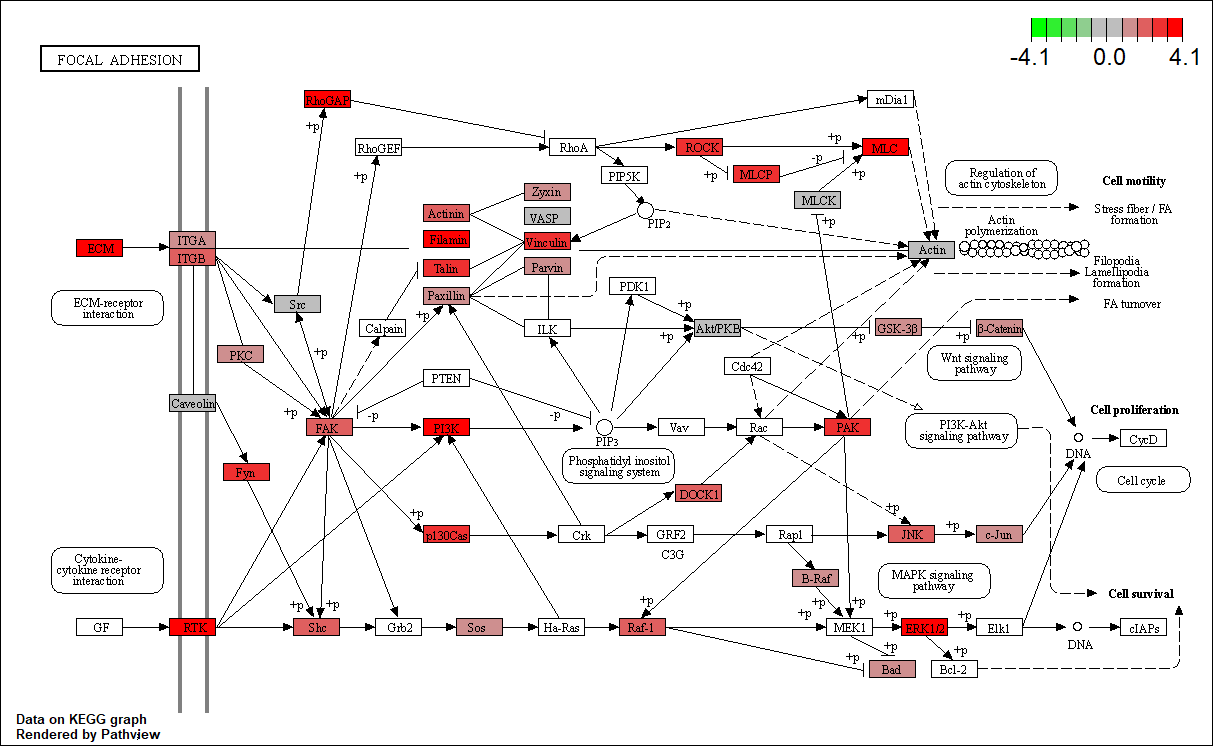


## Figure S10 Phosphorylated tyrosine/serine/threonine related genes in ErbB signaling pathway

The KEGG pathway enrichment results of genes from our mass spectrum data in ErbB signaling pathway. All colored rectangles represent genes corresponding to phosphorylated polypeptide proteins enriched in our mass spectrometry analysis. Red represents increased phosphorylation of this protein in tumor tissue, and green represents decreased phosphorylation of this protein in tumor tissue. This pathway includes ERBB1, ERBB2, ERBB3, and ERBB4, which could affect the proliferation of pancreatic cancer and non-small cell lung cancer, the differentiation of glioma and endometrial cancer.


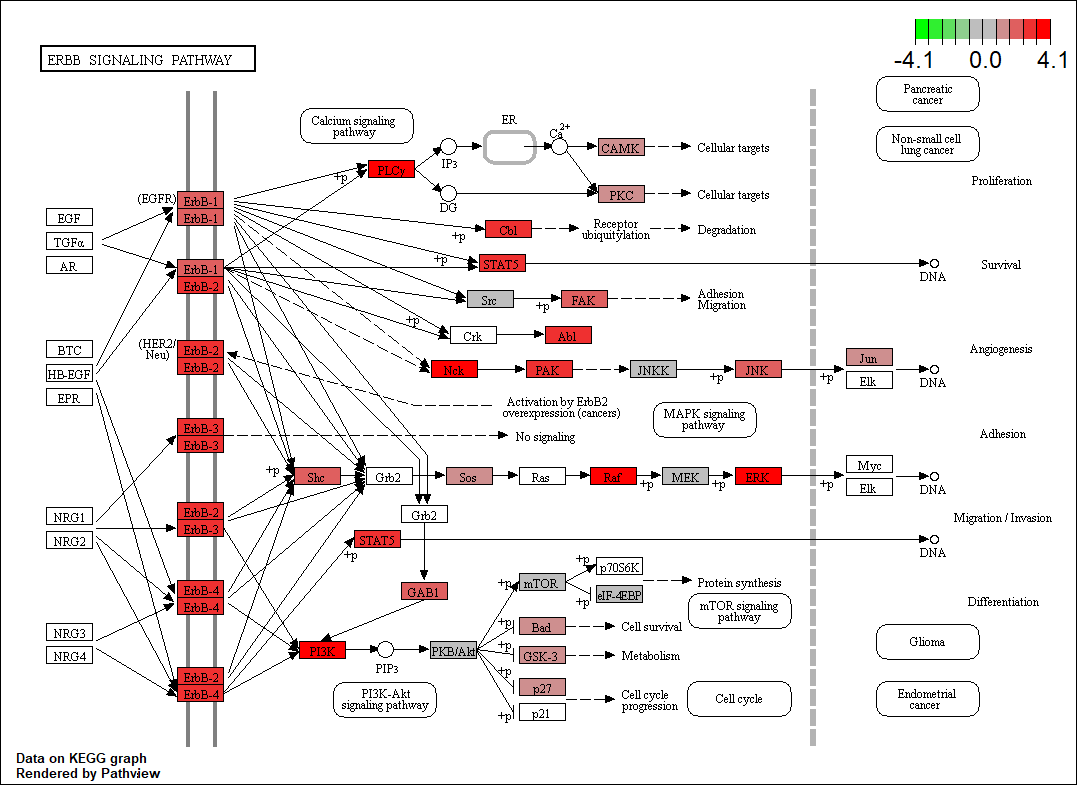


## Figure S11 Phosphorylated tyrosine/serine/threonine related genes in pathway of tight junction

The KEGG pathway enrichment results of genes from our mass spectrum data in pathway of tight junction. All colored rectangles represent genes corresponding to phosphorylated polypeptide proteins enriched in our mass spectrometry analysis. Red represents increased phosphorylation of this protein in tumor tissue, and green represents decreased phosphorylation of this protein in tumor tissue. This pathway includes claudin, occluding, JAM, bves, which could affect the cell polarity, cell proliferation, cell survival, cell differentiation, cell migration.


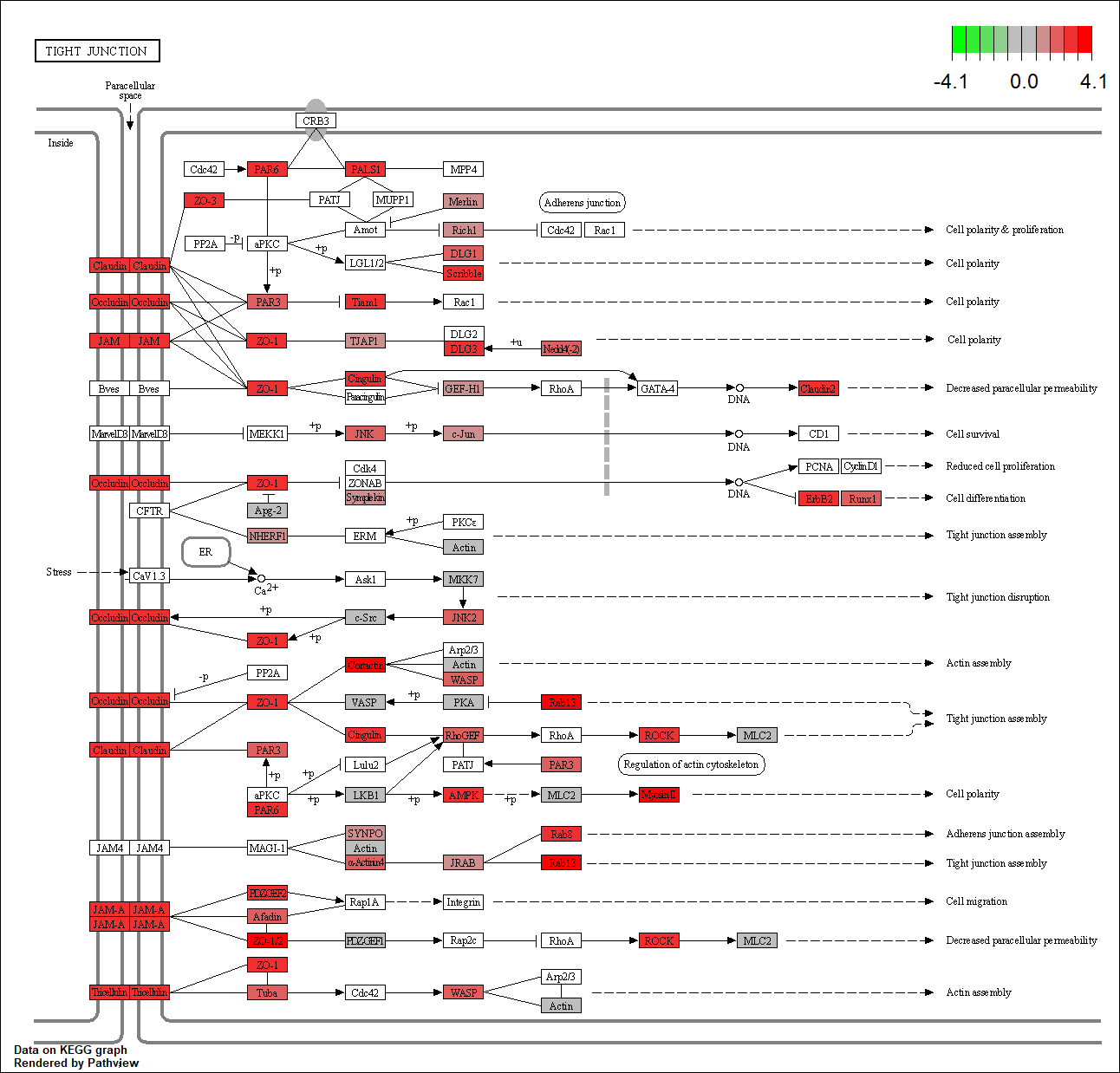


## Figure S12 Phosphorylated tyrosine/serine/threonine related genes in pathway of adherens junction

The KEGG pathway enrichment results of genes from our mass spectrum data in pathway of adherens junction. All colored rectangles represent genes corresponding to phosphorylated polypeptide proteins enriched in our mass spectrometry analysis. Red represents increased phosphorylation of this protein in tumor tissue, and green represents decreased phosphorylation of this protein in tumor tissue. This pathway includes nectin (strong adhesion), cadherin (weak adhesion) et al.


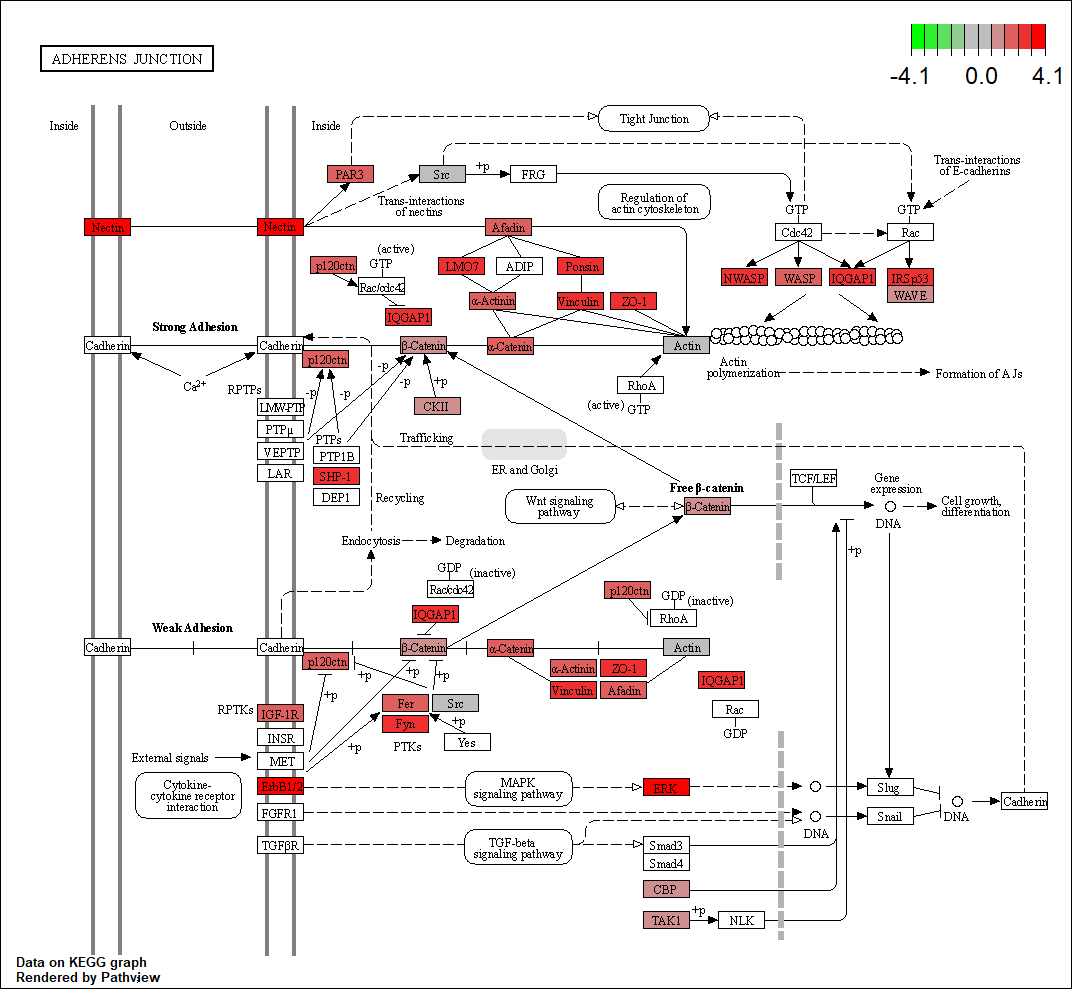


## Figure S13 Phosphorylated tyrosine/serine/threonine related genes in MAPK signaling pathway

The KEGG pathway enrichment results of genes from our mass spectrum data in MAPK signaling pathway. All colored rectangles represent genes corresponding to phosphorylated polypeptide proteins enriched in our mass spectrometry analysis. Red represents increased phosphorylation of this protein in tumor tissue, and green represents decreased phosphorylation of this protein in tumor tissue. This pathway includes classical MAP kinase pathway, JNK and p38 MAP kinase pathway, and ERK5 pathway, which could affect the cell proliferation, cell cycle, cell differentiation, and cell inflammation.


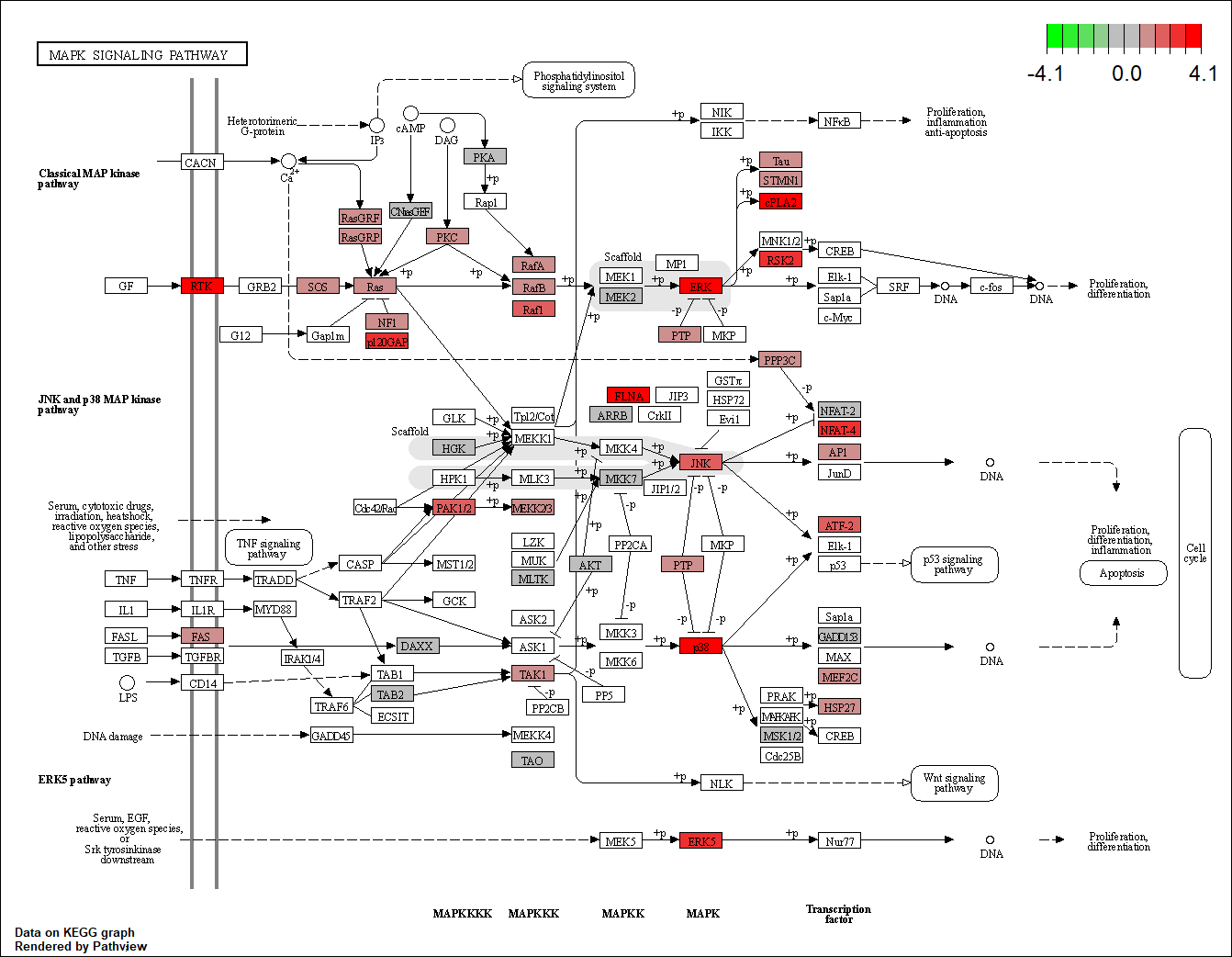


## Figure S14 Survival analysis of patients with breast cancer in different PRKCD level

(A) Kaplan-Meier plot of disease specific survival of patients with breast cancer in different PRKCD level (DATASET: GSE3494-GPL96). (B) Kaplan-Meier plot of distant metastasis free survival of patients with breast cancer in different PRKCD level (DATASET: GSE9195). HR, Hazard Ratio.


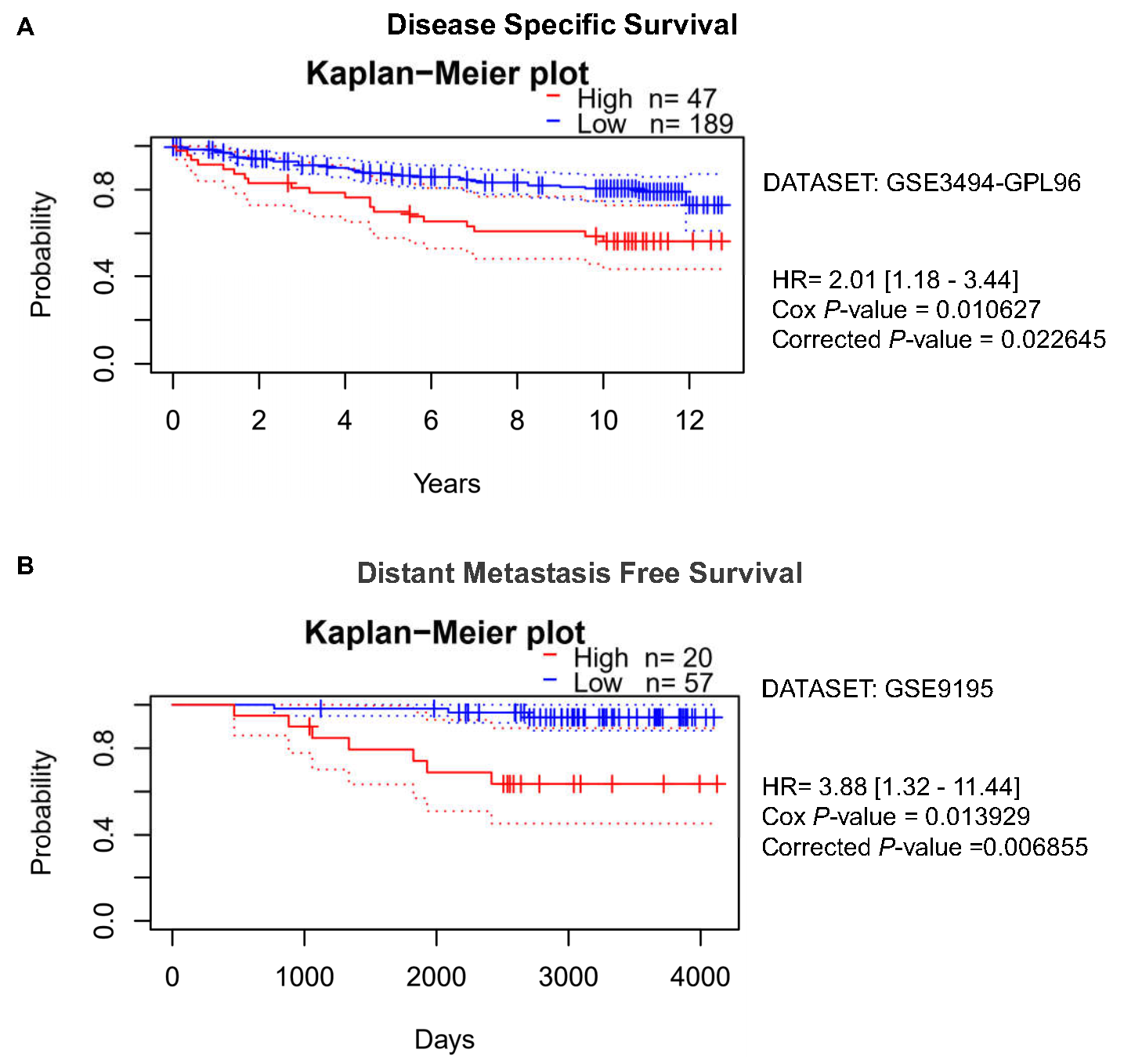


## Figure S15 PRKCD_pY313 promotes malignant biological behaviors of triple-negative breast cancer cells

(A) The knockdown and knockout rate of PRKCD and the PRKCD_pY313 level in MDA-MB-231 stable transfected cell lines. (B) Apoptosis rate of stable transfected MDA-MB-231 and BT549 cell lines with different PRKCD_pY313 level, detected by flow cytometry. (C) The colony formation rate of MDA-MB-231 and BT549 cell lines with different PRKCD_pY313 level. (D) The wound healing assay of stable MDA-MB-231 and BT549 cell lines with different PRKCD_pY313 level. (E) Invasion assay of stable transfected MDA-MB-231 and BT549 cell lines with different PRKCD_pY313 level. OE, overexpression; KD, knockdown. KO, knockout. Mean ± SD, n=3 per group, * *P* < 0.05, ** *P* < 0.01, *** *P* < 0.001, **** *P* < 0.0001.


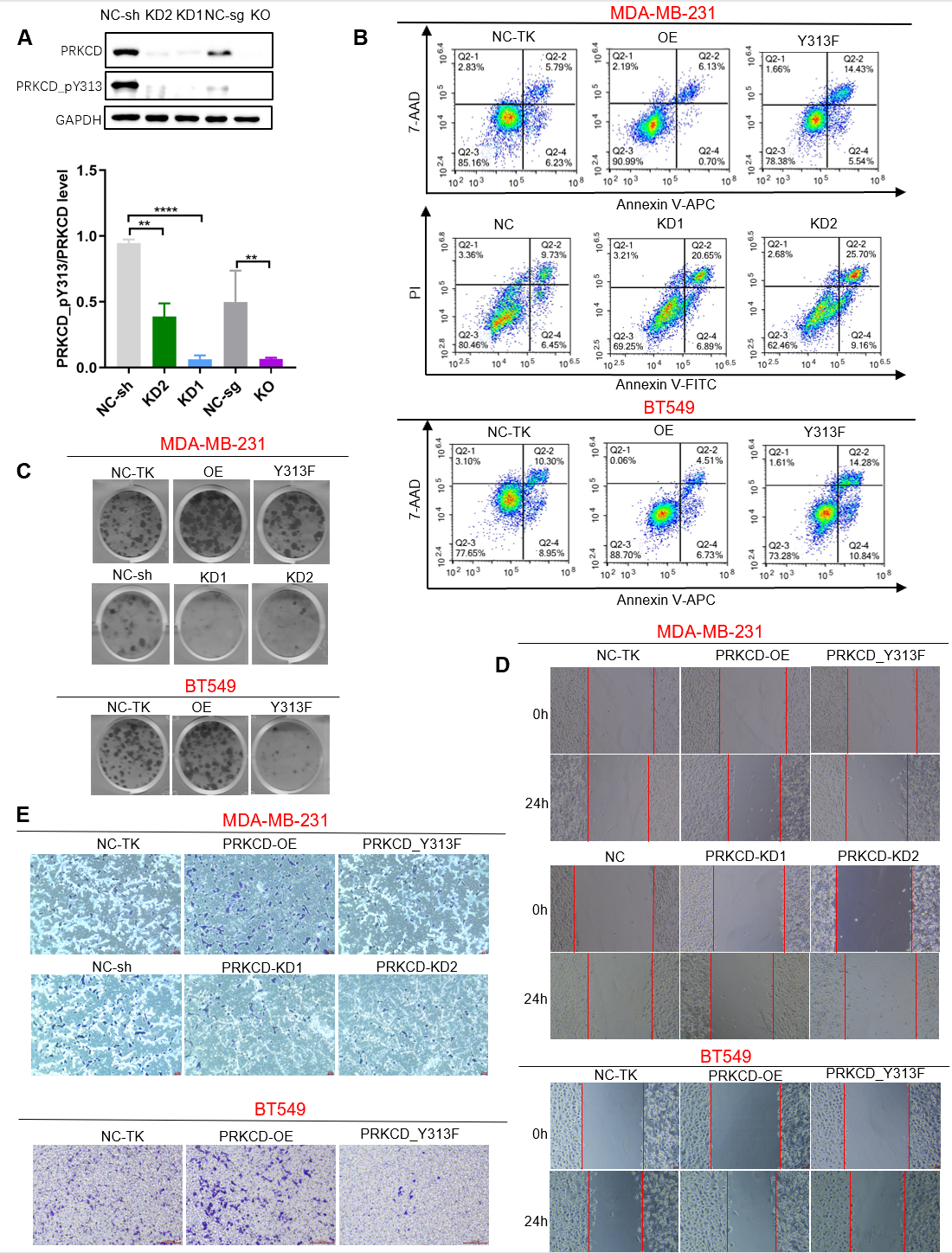


## Figure S16 Expression levels of proteins involved in apoptosis and invasion and metastasis of MDA-MB-231 and BT549 cell lines

(A) Quantification of Figure 5F, apoptotic proteins in MDA-MB-231 cell lines with overexpressed PRKCD_pY313 or PRKCD_Y313F. (B) Quantification of Figure 5F, apoptotic proteins in MDA-MB-231 cell lines with low PRKCD_pY313 level. (C) Quantification of Figure 5F, apoptotic proteins in BT549 cell lines with overexpressed PRKCD_pY313 or PRKCD_Y313F. (D) Quantification of Figure 5F, proteins that mark metastatic capacity in MDA-MB-231 cell lines with overexpressed PRKCD_pY313 or PRKCD_Y313F level. (E) Quantification of Figure 5F, Proteins that mark metastatic capacity in MDA-MB-231 cell lines with low PRKCD_pY313 level. (F) Quantification of Figure 5F, Proteins that mark metastatic capacity in BT549 cell lines with overexpressed PRKCD_pY313 or PRKCD_Y313F level. Mean ± SD, n=3 per group, * *P* < 0.05, ** *P* < 0.01, *** *P* < 0.001, **** *P* < 0.0001.


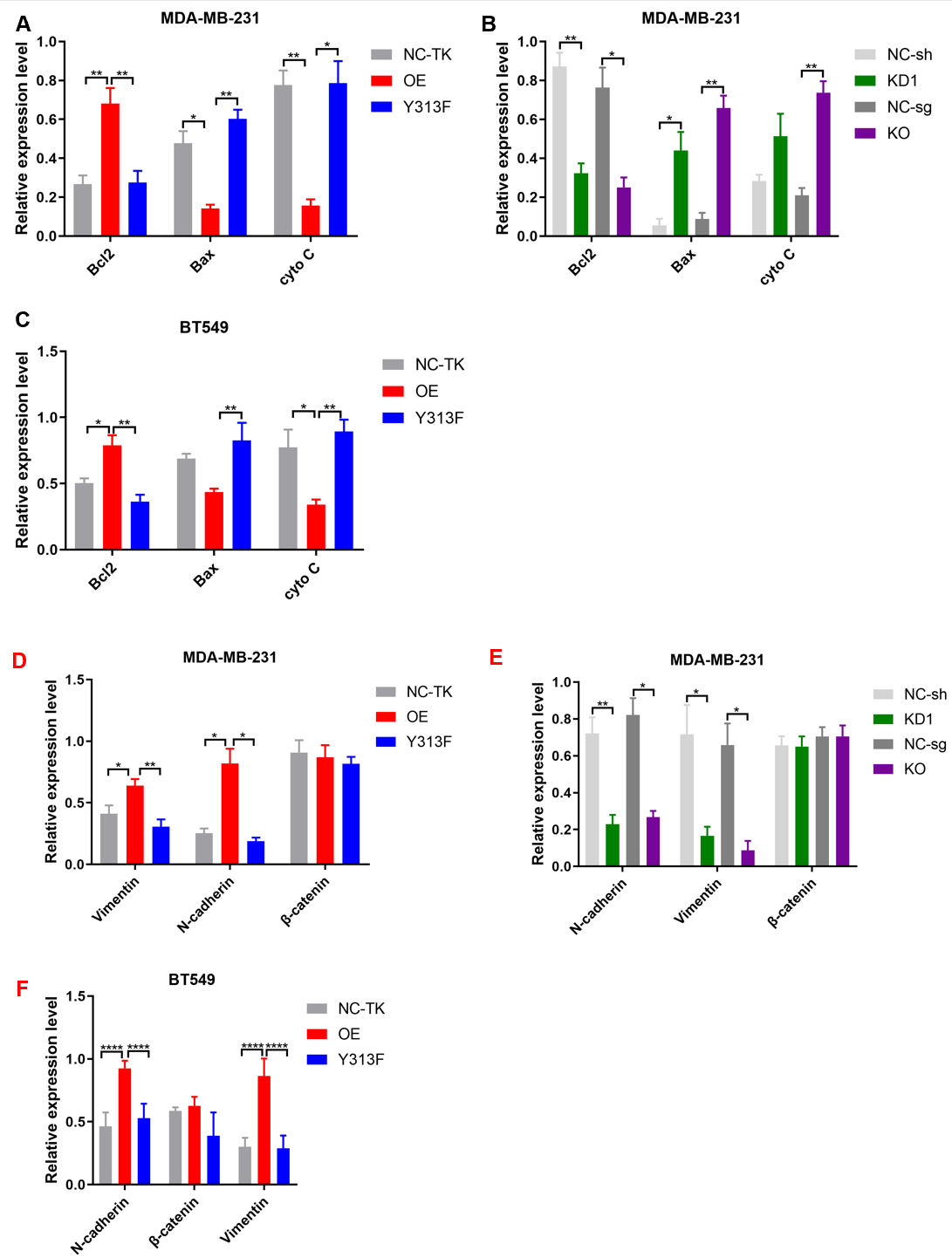


## Figure S17 Proteins and phosphorylated levels of PRKCD-related kinases in MDA-MB-231 and BT549 cell lines with different PRKCD_pY313 level

(A) Quantification of Figure 7A. (B) Quantification of Figure 7B.(C) Quantification of Figure 7C. Mean ± SD, n=3 per group, * *P* < 0.05, ** *P* < 0.01, *** *P* < 0.001, **** *P* < 0.0001.


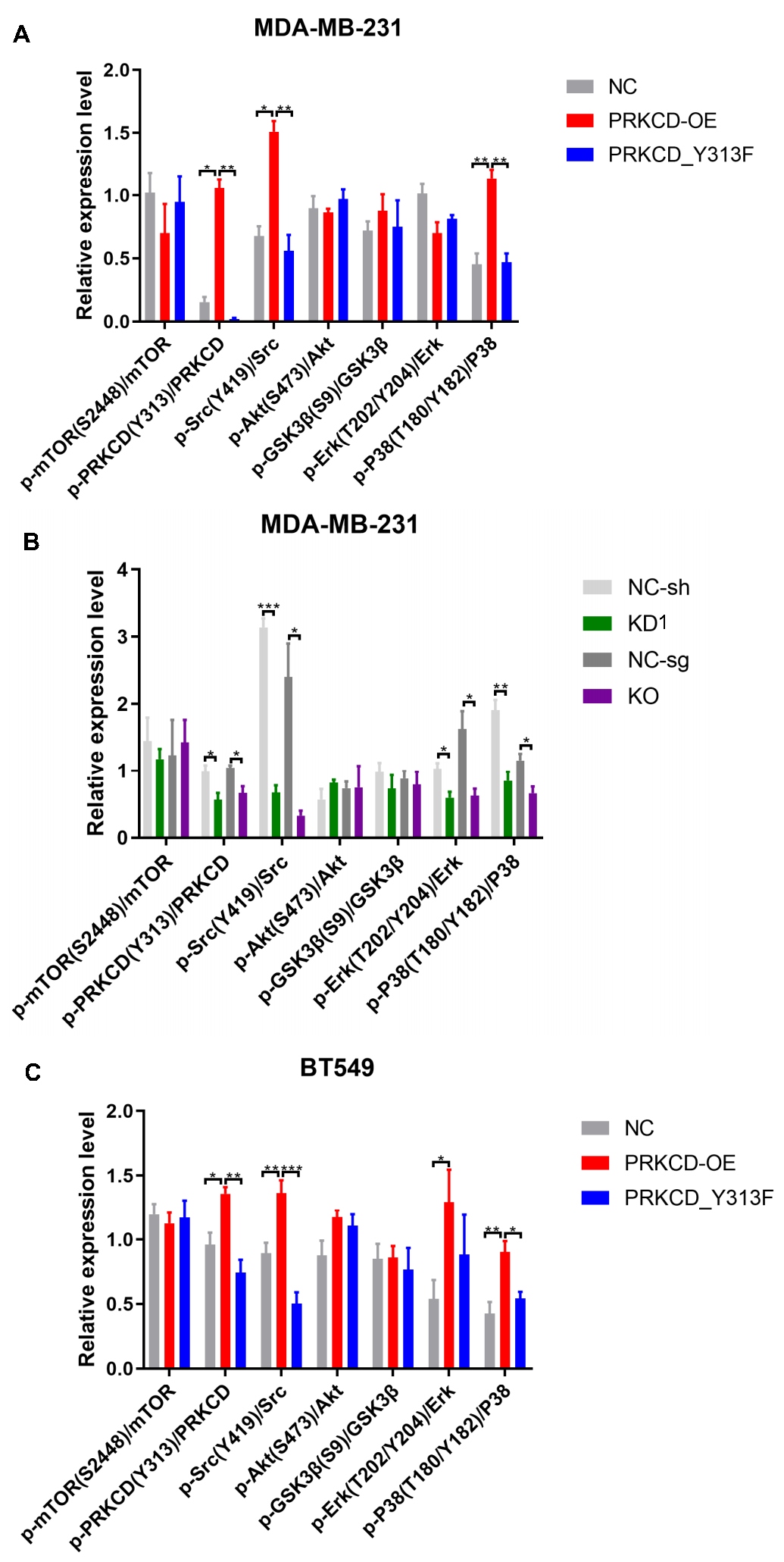


## Figure S18 Proteins and phosphorylated levels of PRKCD-related kinases in MDA-MB-231 and BT549 cell lines with dasatinib treatment

(A) Quantification of Src_pY419/Src level in MDA-MB-231 cell lines in Figure 7D. (B) Quantification of kinases’ activity in MDA-MB-231 cell lines in Figure 7D. (C) Quantification of Src_pY419/Src level in BT549 cell lines in Figure 7E. (D) Quantification of kinases’ activity in BT549 cell lines in Figure 7E. Mean ± SD, n=3 per group, * *P* < 0.05, ** *P* < 0.01, *** *P* < 0.001, **** *P* < 0.0001.


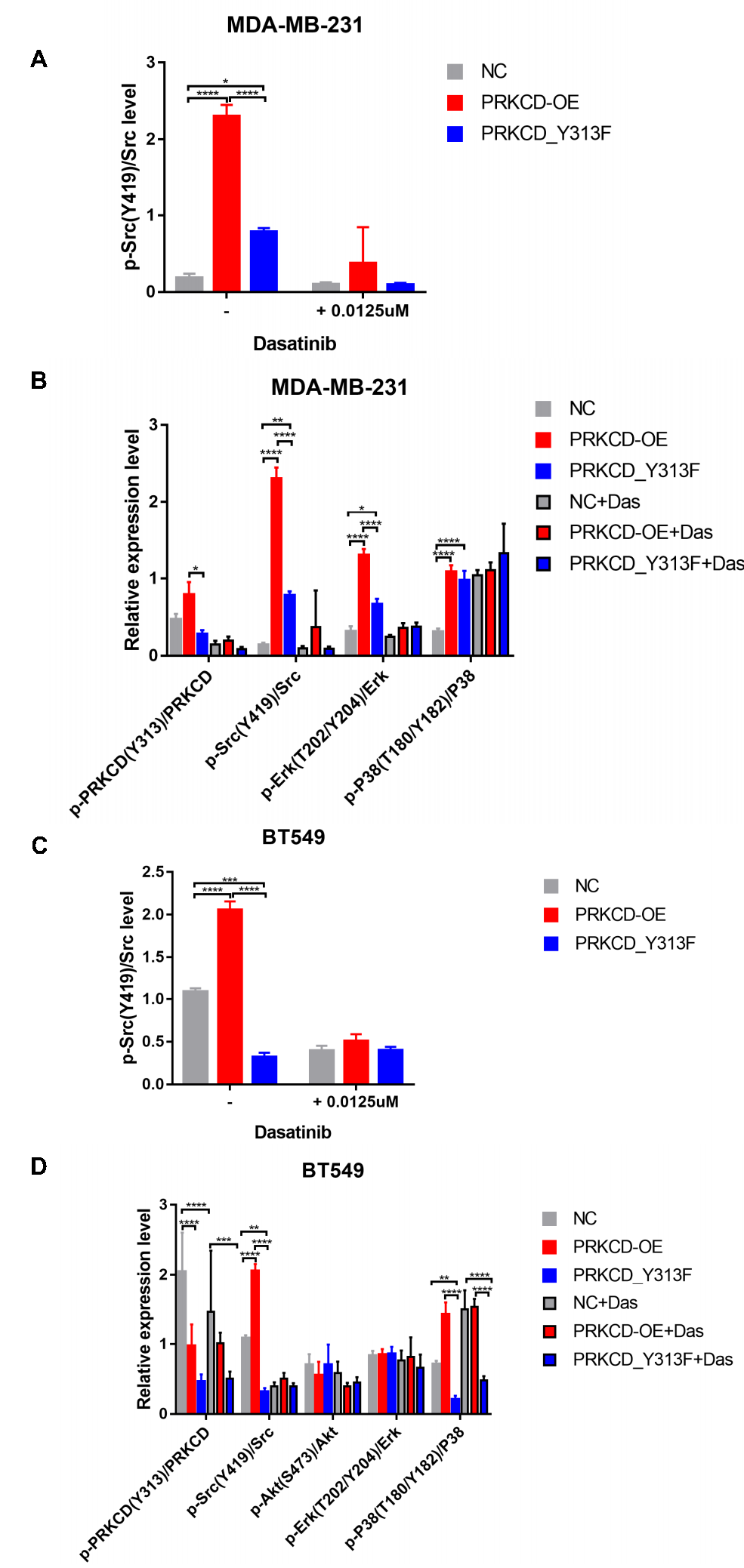


## Figure S19 The MTT assay of stable MDA-MB-231 and BT549 cells treated with dasatinib

(A) The cell relative viability of MDA-MB-231 cell with dasatinib treatment and IC50 (Half maximal inhibitory concentration). (B) The cell relative viability of BT549 cell with dasatinib treatment and IC50. (C) The cell relative viability of MDA-MB-231 cell with 0.01 μM dasatinib treatment. (D) The cell relative viability of BT549 cell with 0.01 μM dasatinib treatment. (E) The cell relative viability of MDA-MB-231 cell with 0.025 μM dasatinib treatment. (F) The cell relative viability of BT549 cell with 0.025 μM dasatinib treatment. (G) The cell relative viability of MDA-MB-231 cell with 0.05 μM dasatinib treatment. (H) The cell relative viability of BT549 cell with 0.05 μM dasatinib treatment. (I) The cell relative viability of MDA-MB-231 cell with 0.1 μM dasatinib treatment. (J) The cell relative viability of BT549 cell with 0.1 μM dasatinib treatment. (K) The cell relative viability of MDA-MB-231 cell with 0.2 μM dasatinib treatment. (L) The cell relative viability of BT549 cell with 0.2 μM dasatinib treatment. Mean ± SD, n=3 per group, * *P* < 0.05, ** *P* < 0.01, *** *P* < 0.001, **** *P* < 0.0001.


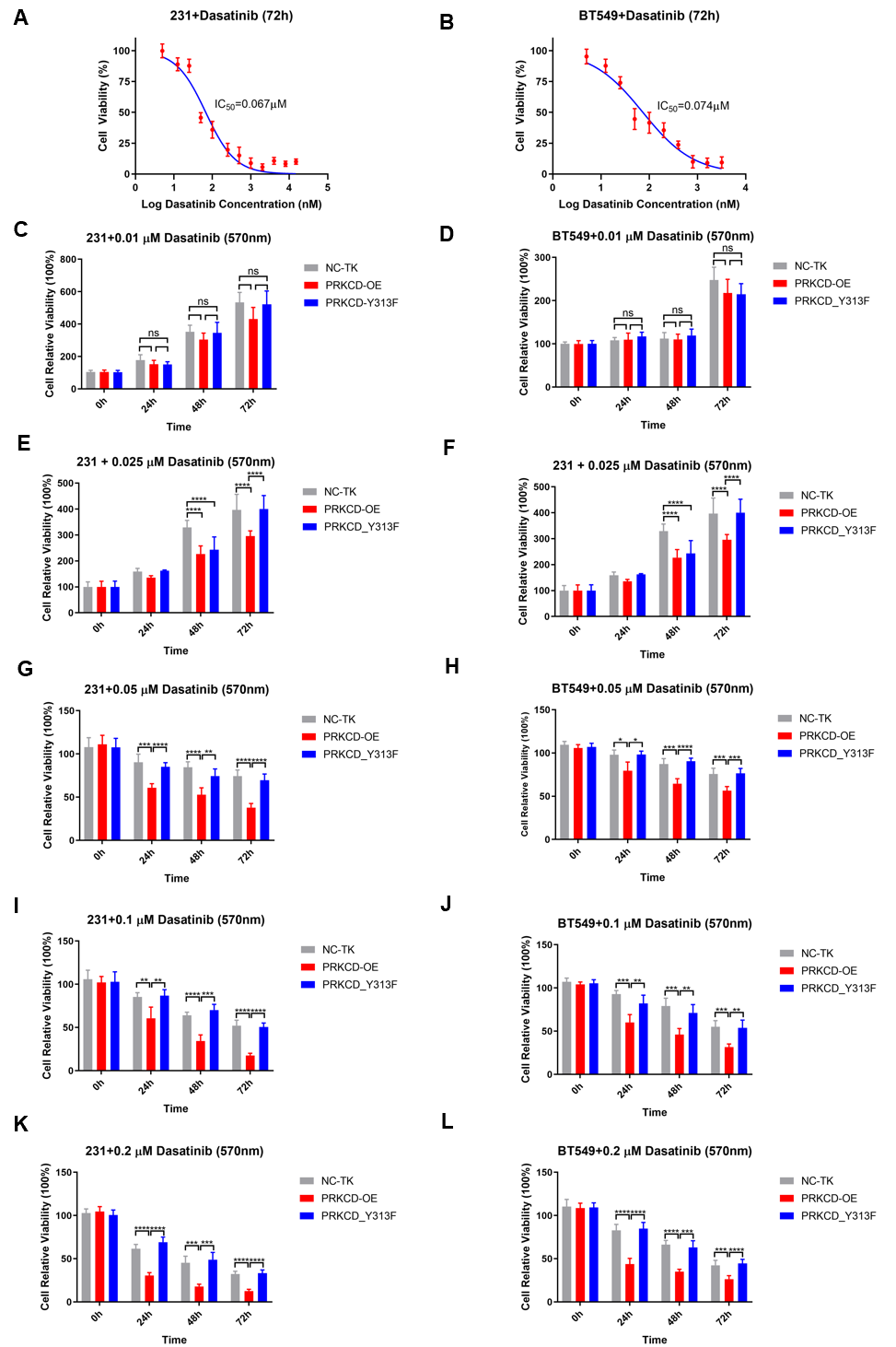

Supplement: Supplementary file 1 — Additional file 1: Table S1. PRKCD knockout-sgRNA sequences. Table S2. PRKCD knockdown shRNA primers. Table S3. PRKCD overexpression and Y313F mutation amplification primers. Table S4. Realtime RT-PCR primers. Table S5. The primary antibodies used in western blot analysis. Fig. S1. Functional enrichment analysis of significant serine/threonine peptides in breast cancer tissues. (A) Barplot of Gene Ontology analysis of significant serine/threonine peptides peptides related genes. (B) Barplot of top 10 KEGG pathways of significant serine/threonine peptides peptides related genes. KEGG, Kyoto Encyclopedia of Genes and Genomes. Fig. S2. Personalized kinase activity map of patient with Luminal A subtype of breast cancer. Each protein frame is divided into four small squares, each of which represents one pY, pS or pT site. The color represents the log10(Ca/N) value, red represents the upregulation of this site, and the darker the color, the higher the upregulation ratio. Blue indicates downregulation, darker color indicates higher downregulation, and gray indicates no such site. The color of the border of each protein bar represents the sum of the log10(Ca/N) values of all sites of the protein. The thickness of the lines between the proteins indicates a test score ranging from 1 to 10, and the color of the lines ranging from gray to red indicates a test score ranging from 0 to 1. Ca/N, ratio of expression levels in breast cancer tumors to that in normal breast tissue. Fig. S3. Personalized kinase activity map of patient with Luminal B subtype of breast cancer. Each protein frame is divided into four small squares, each of which represents one pY, pS or pT site. The color represents the log10(Ca/N) value, red represents the upregulation of this site, and the darker the color, the higher the upregulation ratio. Blue indicates downregulation, darker color indicates higher downregulation, and gray indicates no such site. The color of the border of each protein bar represents [file 12964_2024_1487_MOESM1_ESM.docx]
